# Supplementary material for: A fungal sesquiterpene biosynthesis gene cluster critical for mutualist-pathogen transition in Colletotrichum tofieldiae
Source: Nat Commun. 2023 Sep 6;14:5288. doi: 10.1038/s41467-023-40867-w (PMC10482981; doi:10.1038/s41467-023-40867-w)
Supplement: Supplementary file 1 — Supplementary Information [file 41467_2023_40867_MOESM1_ESM.pdf]

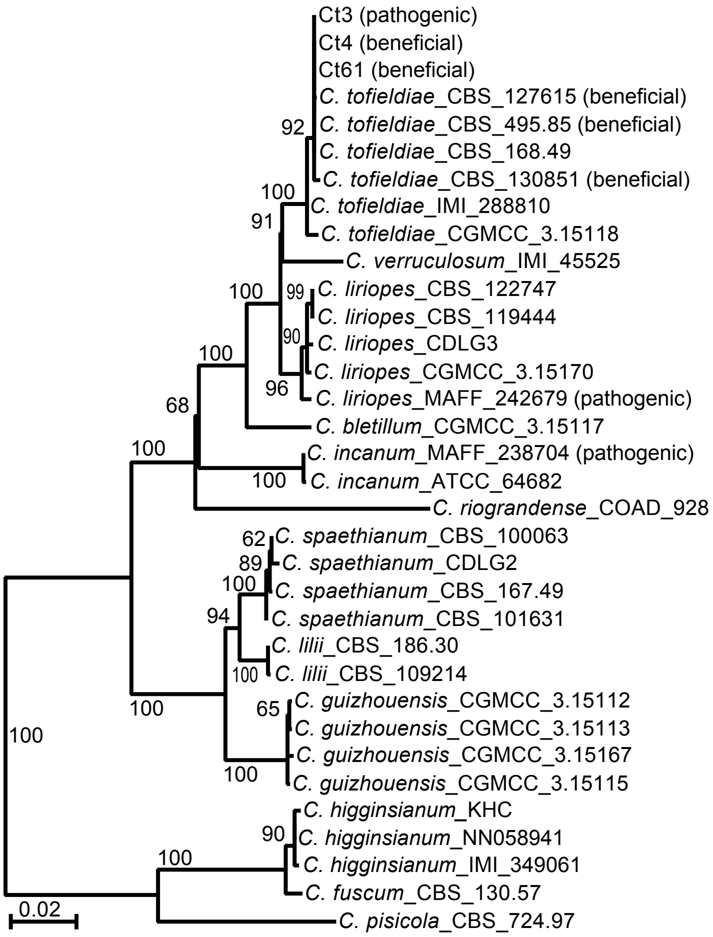

Supplementary Figure 1: Ct3 and Ct4 belong to *Colletotrichum tofieldiae* species.

Maximum-likelihood phylogenetic tree of 34 strains in 12 species of the genus *Colletotrichum* based on a concatenated alignment of six fungal marker nucleotide sequences (ITS, TUB2, ACT, HIS3, CHS-1, and GAPDH). Ultrafast bootstrap values (1,000 replicates) are shown on the branches. The strains in the *C. destructivum* species complex are used as an outgroup. Lifestyles of some *Colletotrichum* fungi in *A. thaliana* roots under low Pi were indicated (beneficial or pathogenic). CBS_127615 = Ct127, CBS_495.85 = Ct49, CBS_130851 =Ct130.

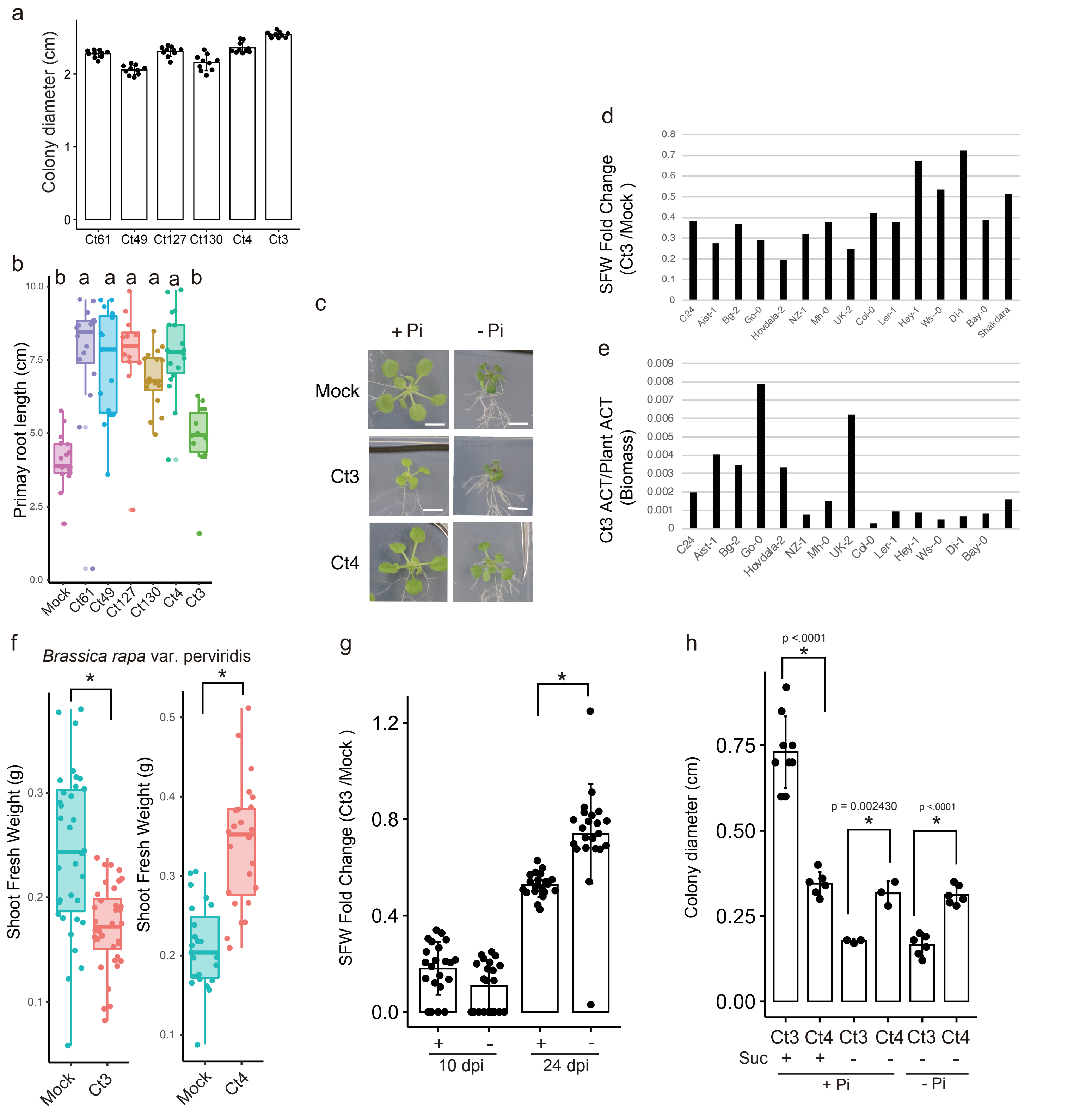

Supplementary Figure 2: A Ct strain (Ct3) inhibits plant growth of *A. thaliana* and *B. rapa*. **a** Measurements of fungal colony diameters (n = 10). Bars = ±SD. **b** Measurements of *A. thaliana* primary root length upon fungal inoculation. Plants were incubated for 24 days under low-Pi (50 μM KH_2PO_4) from germination either with mock treatment, Ct61, Ct49, Ct127, Ct130, Ct4 or Ct3 (Mock: n=17, Ct61: n=16, Ct49: n=15, Ct127: n=12, Ct130: n=16, Ct4: n=18, Ct3: n=14). Each dot represents an individual plant sample. Different letters indicate significantly different statistical groups (ANOVA, Tukey-HSD test, $p < 0.05$). **c** Pictures of *A. thaliana* plants grown in normal or low Pi conditions with and without Ct3 or Ct4. The picture was taken after 24-day post inoculation (24 dpi) on the same scale. bars = 0.5 cm. **d, e** Inoculation of Ct3 against 15 different *A. thaliana* accessions under low Pi. The negative impacts on plant growth by Ct3 were estimated by SFW fold change (Ct3/Mock) at 24 dpi. At least four plants per accession and treatment were used to calculate SFW fold change. qRT-PCR analyses were used to measure fungal biomass as estimated by relative expression between CtACTIN and PlantACTIN (*CtACTIN*/*PlantACTIN*). **f** Measurement of *B. rapa* SFW either upon Ct3 (Left, Mock: n=34, Ct3: n=36) or Ct4 (Right, Mock: n=23, Ct4: n=24) inoculation under low nutrient soils. Asterisks indicate significantly different statistical groups ($p < .0001$, two tailed t-test). **g** Degree of Ct3-mediated plant growth inhibition under normal (+, 625 μM KH_2PO_4) and low (-) Pi conditions. Asterisks indicate significantly different statistical groups ($p < .0001$, two tailed t-test, Bars = ±SD). **h** Fungal growth either on normal Pi + 0.8% sucrose, normal Pi without sucrose, or low Pi without sucrose media. The diameter of each fungal colony was measured after 3 days of incubation. Asterisks indicate significantly different values between Ct3 and Ct4 (±SD, n = 3~9, $p < 0.01$, two tailed t-test).

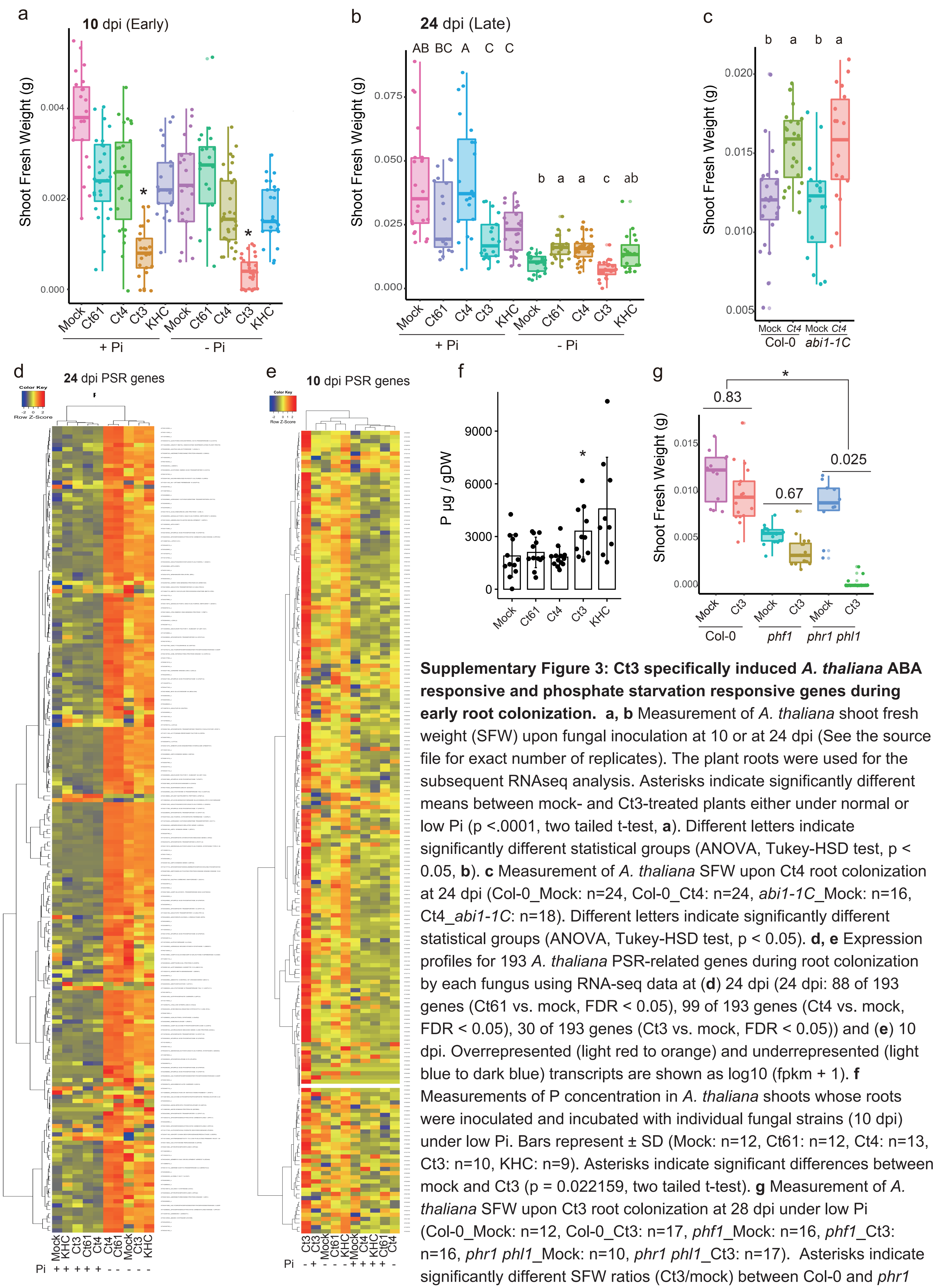

Supplementary Figure 3: Ct3 specifically induced *A. thaliana* ABA responsive and phosphate starvation responsive genes during early root colonization. **a, b** Measurement of *A. thaliana* shoot fresh weight (SFW) upon fungal inoculation at 10 or at 24 dpi (See the source file for exact number of replicates). The plant roots were used for the subsequent RNAseq analysis. Asterisks indicate significantly different means between mock- and Ct3-treated plants either under normal or low Pi ($p < .0001$, two tailed t-test, **a**). Different letters indicate significantly different statistical groups (ANOVA, Tukey-HSD test, $p < 0.05$, **b**). **c** Measurement of *A. thaliana* SFW upon Ct4 root colonization at 24 dpi (Col-0_Mock: n=24, Col-0_Ct4: n=24, *abi1-1C*_Mock: n=16, Ct4_*abi1-1C*: n=18). Different letters indicate significantly different statistical groups (ANOVA, Tukey-HSD test, $p < 0.05$). **d, e** Expression profiles for 193 *A. thaliana* PSR-related genes during root colonization by each fungus using RNA-seq data at (**d**) 24 dpi (24 dpi: 88 of 193 genes (Ct61 vs. mock, FDR < 0.05), 99 of 193 genes (Ct4 vs. mock, FDR < 0.05), 30 of 193 genes (Ct3 vs. mock, FDR < 0.05)) and (**e**) 10 dpi. Overrepresented (light red to orange) and underrepresented (light blue to dark blue) transcripts are shown as log10 (fpkm + 1). **f** Measurements of P concentration in *A. thaliana* shoots whose roots were inoculated and incubated with individual fungal strains (10 dpi) under low Pi. Bars represent ± SD (Mock: n=12, Ct61: n=12, Ct4: n=13, Ct3: n=10, KHC: n=9). Asterisks indicate significant differences between mock and Ct3 ($p = 0.022159$, two tailed t-test). **g** Measurement of *A. thaliana* SFW upon Ct3 root colonization at 28 dpi under low Pi (Col-0_Mock: n=12, Col-0_Ct3: n=17, *phf1*_Mock: n=16, *phf1*_Ct3: n=16, *phr1 phl1*_Mock: n=10, *phr1 phl1*_Ct3: n=17). Asterisks indicate significantly different SFW ratios (Ct3/mock) between Col-0 and *phr1 phl1* ($p < 0.05$, two tailed t-test).

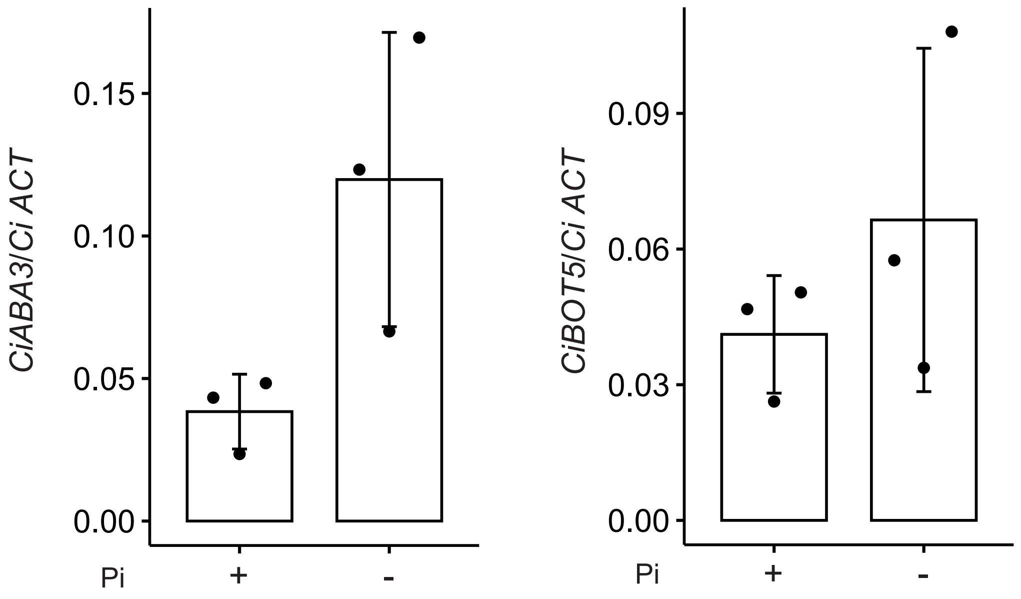

Supplementary Figure 4a: Fungal ABA and BOT biosynthesis genes were induced during root colonization by *C. incanum*. The expression of *CiABA3* or *CiBOT5* genes during root colonization by *C. incanum*. Plants were incubated with *C. incanum* for 23 dpi under normal (+) or low (-) Pi (±SD, n =3).

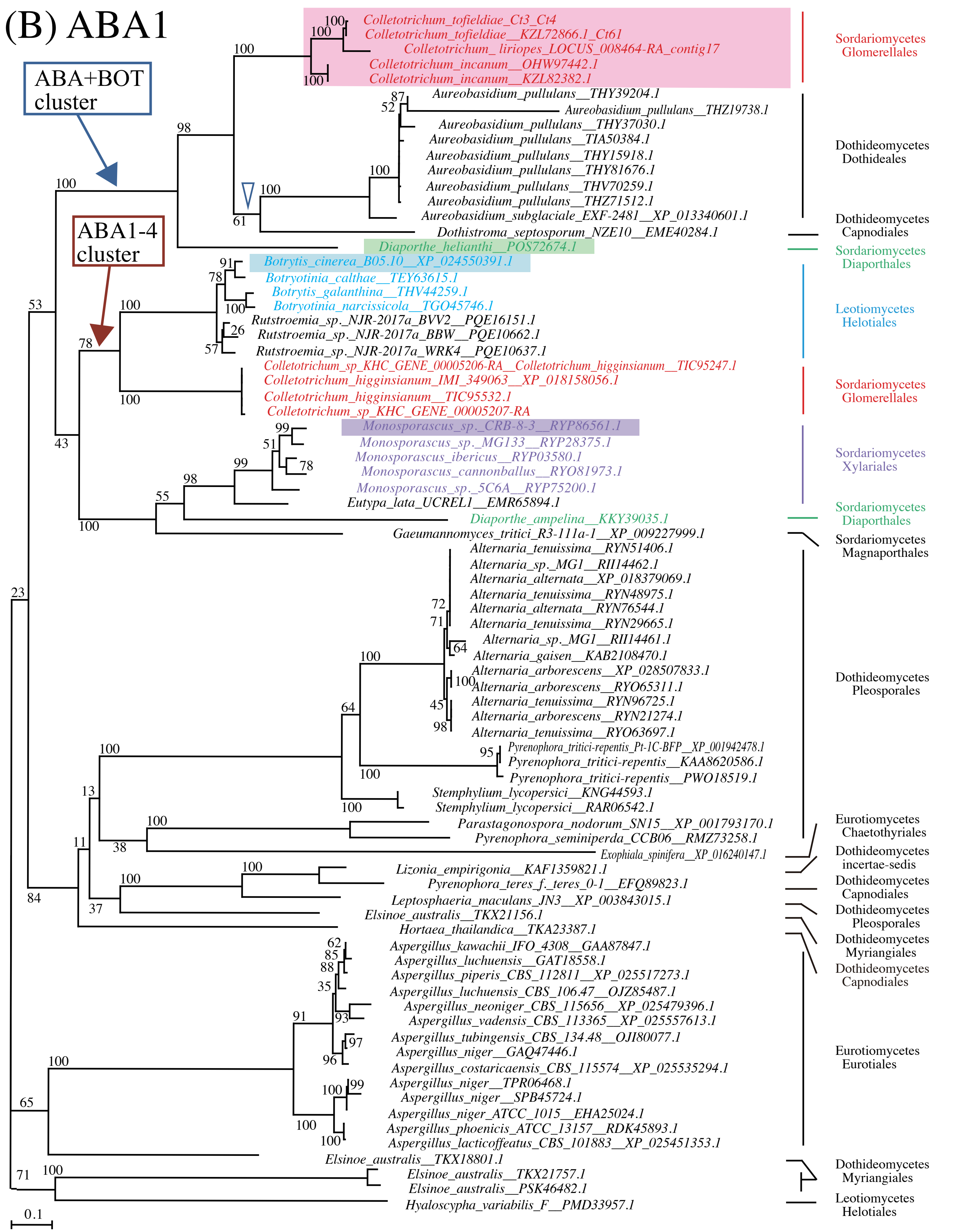

b-l. Phylogenies of ABA and BOT genes using IQ-TREE version 1.6.11. Arrow indicates origins of various gene clusters. Triangles with the same color as the arrows show deletion of the clusters. Genes of *Colletotrichum*, *Botrytis*, *Diaporthe*, and *Monosporascus* are indicated with the colors of red, blue, green, and purple, respectively. Organism strains that have both ABA and BOT gene clusters in their genome are labeled with the same color background as the letters. The bootstrap probability is shown on the branches. The scale bar represents substitutions per site. **b-e** ABA1-4 genes. **f-l** BOT1-7 genes.

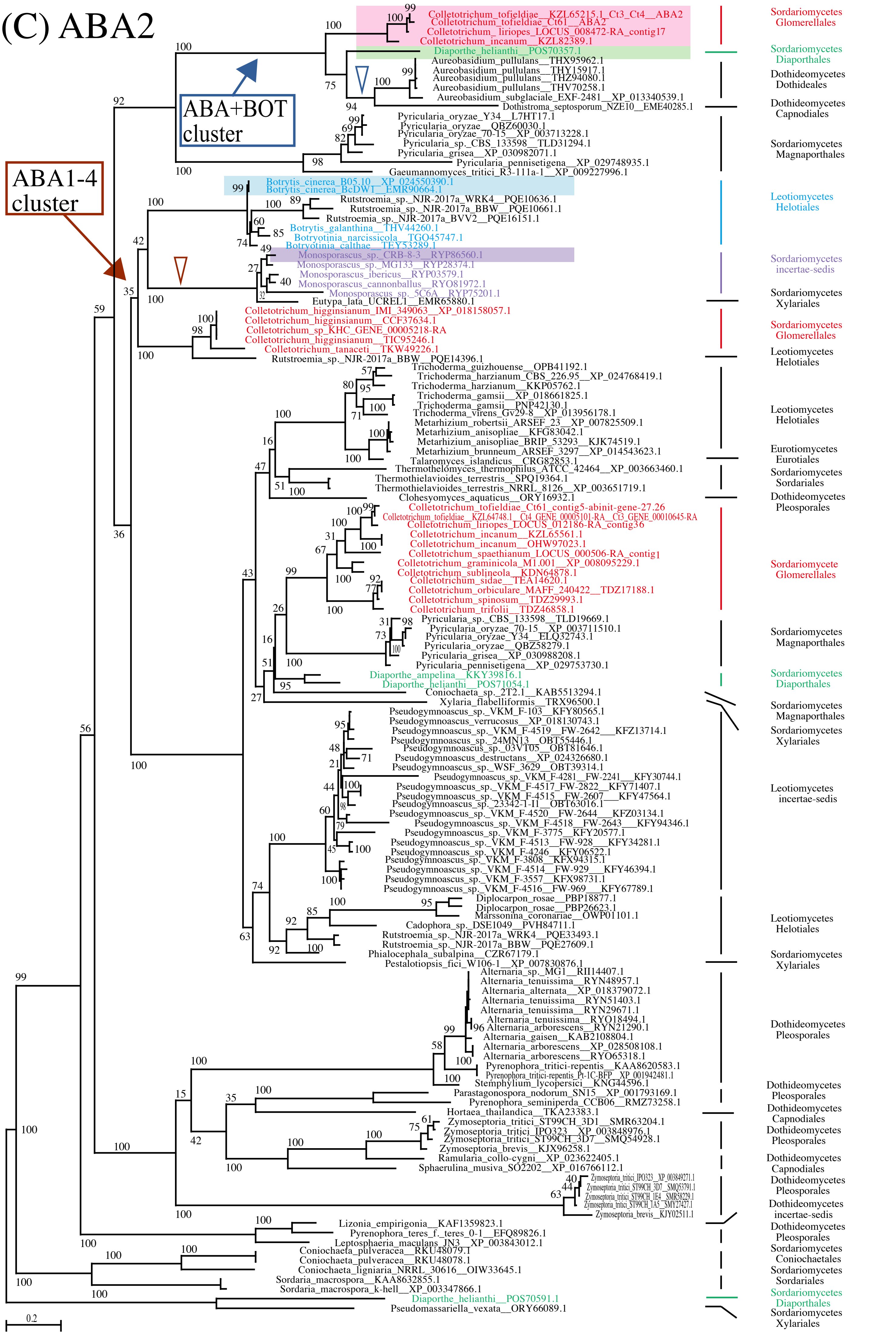

(C) ABA2
ABA+BOT cluster
ABA1-4 cluster
Colletotrichum_tofieldiae__KZL65215.1_Ct3_Ct4__ABA2
Colletotrichum_tofieldiae_Ct61__ABA2
Colletotrichum_liriopes_LOCUS_008472-RA_contig17
Colletotrichum_incanum__KZL82389.1
Diaporthe_helianthi__POS70357.1
Aureobasidium_pullulans__THX95962.1
Aureobasidium_pullulans__THY15917.1
Aureobasidium_pullulans__THZ94080.1
Aureobasidium_pullulans__THV70258.1
Aureobasidium_subglaciale_EXF-2481__XP_013340539.1
Dothistroma_septosporum_NZE10__EME40285.1
Pyricularia_oryzae_Y34__L7HT17.1
Pyricularia_oryzae__QBZ60030.1
Pyricularia_oryzae_70-15__XP_003713228.1
Pyricularia_sp._CBS_133598__TLD31294.1
Pyricularia_grisea__XP_030982071.1
Pyricularia_pennisetigena__XP_029748935.1
Gaeumannomyces_tritici_R3-111a-1__XP_009227996.1
Botrytis_cinerea_B05.10__XP_024550390.1
Botrytis_cinerea_BcDW1__EMR90664.1
Rutstroemia_sp._NJR-2017a_WRK4__PQE10636.1
Rutstroemia_sp._NJR-2017a_BBW__PQE10661.1
Rutstroemia_sp._NJR-2017a_BVV2__PQE16151.1
Botrytis_galanthina__THV44260.1
Botryotinia_narcissicola__TGO45747.1
Botryotinia_calthae__TEY53289.1
Monosporascus_sp._CRB-8-3__RYP86560.1
Monosporascus_sp._MG133__RYP28374.1
Monosporascus_ibericus__RYP03579.1
Monosporascus_cannonballus__RYO81972.1
Monosporascus_sp._5C6A__RYP75201.1
Eutypa_lata_UCREL1__EMR65880.1
Colletotrichum_higginsianum_IMI_349063__XP_018158057.1
Colletotrichum_higginsianum__CCF37634.1
Colletotrichum_sp_KHC_GENE_00005218-RA
Colletotrichum_higginsianum__TIC95246.1
Colletotrichum_tanaceti__TKW49226.1
Rutstroemia_sp._NJR-2017a_BBW__PQE14396.1
Trichoderma_guizhouense__OPB41192.1
Trichoderma_harzianum_CBS_226.95__XP_024768419.1
Trichoderma_harzianum__KKP05762.1
Trichoderma_gamsii__XP_018661825.1
Trichoderma_gamsii__PNP42130.1
Trichoderma_virens_Gv29-8__XP_013956178.1
Metarhizium_robertsii_ARSEF_23__XP_007825509.1
Metarhizium_anisopliae__KFG83042.1
Metarhizium_anisopliae_BRIP_53293__KJK74519.1
Metarhizium_brunneum_ARSEF_3297__XP_014543623.1
Talaromyces_islandicus__CRG82853.1
Thermothelomyces_thermophilus_ATCC_42464__XP_003663460.1
Thermothielavioides_terrestris__SPQ19364.1
Thermothielavioides_terrestris_NRRL_8126__XP_003651719.1
Clohesyomyces_aquaticus__ORY16932.1
Colletotrichum_tofieldiae_Ct61_contig5-abinit-gene-27.26
Colletotrichum_tofieldiae__KZL64748.1__Ct4_GENE_00005101-RA__Ct3_GENE_00010645-RA
Colletotrichum_liriopes_LOCUS_012186-RA_contig36
Colletotrichum_incanum__KZL65561.1
Colletotrichum_incanum__OHW97023.1
Colletotrichum_spaethianum_LOCUS_000506-RA_contig1
Colletotrichum_graminicola_M1.001__XP_008095229.1
Colletotrichum_sublineola__KDN64878.1
Colletotrichum_sidae__TEA14620.1
Colletotrichum_orbiculare_MAFF_240422__TDZ17188.1
Colletotrichum_spinosum__TDZ29993.1
Colletotrichum_trifolii__TDZ46858.1
Pyricularia_sp._CBS_133598__TLD19669.1
Pyricularia_oryzae_70-15__XP_003711510.1
Pyricularia_oryzae_Y34__ELQ32743.1
Pyricularia_oryzae__QBZ58279.1
Pyricularia_grisea__XP_030988208.1
Pyricularia_pennisetigena__XP_029753730.1
Diaporthe_ampelina__KKY39816.1
Diaporthe_helianthi__POS71054.1
Coniochaeta_sp._2T2.1__KAB5513294.1
Xylaria_flabelliformis__TRX96500.1
Pseudogymnoascus_sp._VKM_F-103__KFY80565.1
Pseudogymnoascus_verrucosus__XP_018130743.1
Pseudogymnoascus_sp._VKM_F-4519__FW-2642__KFZ13714.1
Pseudogymnoascus_sp._24MN13__OBT55446.1
Pseudogymnoascus_sp._03VT05__OBT81646.1
Pseudogymnoascus_destructans__XP_024326680.1
Pseudogymnoascus_sp._WSF_3629__OBT39314.1
Pseudogymnoascus_sp._VKM_F-4281__FW-2241__KFY30744.1
Pseudogymnoascus_sp._VKM_F-4517_FW-2822__KFY71407.1
Pseudogymnoascus_sp._VKM_F-4515__FW-2607__KFY47564.1
Pseudogymnoascus_sp._23342-1-I1__OBT63016.1
Pseudogymnoascus_sp._VKM_F-4520__FW-2644__KFZ03134.1
Pseudogymnoascus_sp._VKM_F-4518__FW-2643__KFY94346.1
Pseudogymnoascus_sp._VKM_F-3775__KFY20577.1
Pseudogymnoascus_sp._VKM_F-4513__FW-928__KFY34281.1
Pseudogymnoascus_sp._VKM_F-4246__KFY06522.1
Pseudogymnoascus_sp._VKM_F-3808__KFX94315.1
Pseudogymnoascus_sp._VKM_F-4514__FW-929__KFY46394.1
Pseudogymnoascus_sp._VKM_F-3557__KFX98731.1
Pseudogymnoascus_sp._VKM_F-4516__FW-969__KFY67789.1
Diplocarpon_rosae__PBP18877.1
Diplocarpon_rosae__PBP26623.1
Marssonina_coronariae__OWP01101.1
Cadophora_sp._DSE1049__PVH84711.1
Rutstroemia_sp._NJR-2017a_WRK4__PQE33493.1
Rutstroemia_sp._NJR-2017a_BBW__PQE27609.1
Phialocephala_subalpina__CZR67179.1
Pestalotiopsis_fici_W106-1__XP_007830876.1
Alternaria_sp._MG1__RII14407.1
Alternaria_tenuissima__RYN48957.1
Alternaria_alternata__XP_018379072.1
Alternaria_tenuissima__RYN51403.1
Alternaria_tenuissima__RYN29671.1
Alternaria_tenuissima__RYO18494.1
Alternaria_arborescens__RYN21290.1
Alternaria_gaisen__KAB2108804.1
Alternaria_arborescens__XP_028508108.1
Alternaria_arborescens__RYO65318.1
Pyrenophora_tritici-repentis__KAA8620583.1
Pyrenophora_tritici-repentis_Pt-1C-BFP__XP_001942481.1
Stemphylium_lycopersici__KNG44596.1
Parastagonospora_nodorum_SN15__XP_001793169.1
Pyrenophora_seminiperda_CCB06__RMZ73258.1
Hortaea_thailandica__TKA23383.1
Zymoseptoria_tritici_ST99CH_3D1__SMR63204.1
Zymoseptoria_tritici_IPO323__XP_003848976.1
Zymoseptoria_tritici_ST99CH_3D7__SMQ54928.1
Zymoseptoria_brevis__KJX96258.1
Ramularia_collo-cygni__XP_023622405.1
Sphaerulina_musiva_SO2202__XP_016766112.1
Zymoseptoria_tritici_IPO323__XP_003849271.1
Zymoseptoria_tritici_ST99CH_3D7__SMQ53791.1
Zymoseptoria_tritici_ST99CH_1E4__SMR58229.1
Zymoseptoria_tritici_ST99CH_1A5__SMY27427.1
Zymoseptoria_brevis__KJY02511.1
Lizonia_empirigonia__KAF1359823.1
Pyrenophora_teres_f._teres_0-1__EFQ89826.1
Leptosphaeria_maculans_JN3__XP_003843012.1
Coniochaeta_pulveracea__RKU48079.1
Coniochaeta_pulveracea__RKU48078.1
Coniochaeta_ligniaria_NRRL_30616__OIW33645.1
Sordaria_macrospora__KAA8632855.1
Sordaria_macrospora_k-hell__XP_003347866.1
Diaporthe_helianthi__POS70591.1
Pseudomassariella_vexata__ORY66089.1
0.2
Sordariomycetes Glomerellales
Sordariomycetes Diaporthales
Dothideomycetes Dothideales
Dothideomycetes Capnodiales
Sordariomycetes Magnaporthales
Leotiomycetes Helotiales
Sordariomycetes incertae-sedis
Sordariomycetes Xylariales
Sordariomycetes Glomerellales
Leotiomycetes Helotiales
Leotiomycetes Helotiales
Eurotiomycetes Eurotiales
Sordariomycetes Sordariales
Dothideomycetes Pleosporales
Sordariomycete Glomerellales
Sordariomycetes Magnaporthales
Sordariomycetes Diaporthales
Sordariomycetes Magnaporthales
Sordariomycetes Xylariales
Leotiomycetes incertae-sedis
Leotiomycetes Helotiales
Sordariomycetes Xylariales
Dothideomycetes Pleosporales
Dothideomycetes Pleosporales
Dothideomycetes Capnodiales
Dothideomycetes Pleosporales
Dothideomycetes Capnodiales
Dothideomycetes Pleosporales
Dothideomycetes incertae-sedis
Dothideomycetes Pleosporales
Sordariomycetes Coniochaetales
Sordariomycetes Sordariales
Sordariomycetes Diaporthales
Sordariomycetes Xylariales

(D) ABA3

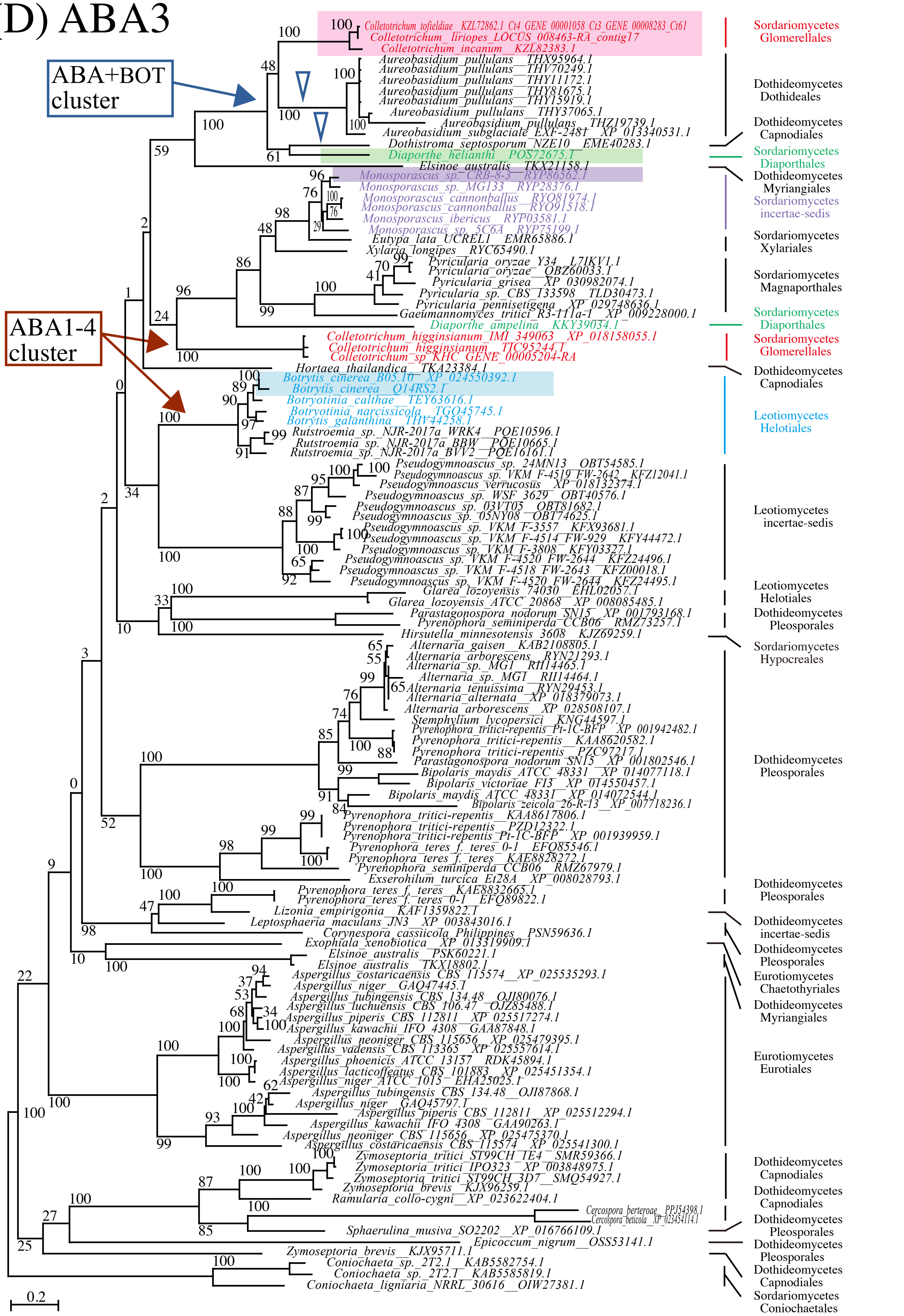
ABA+BOT cluster
ABA1-4 cluster
Colletotrichum_tofieldiae__KZL72862.1_Ct4_GENE_00001058_Ct3_GENE_00008283_Ct61
Colletotrichum_liriopes_LOCUS_008463-RA_contig17
Colletotrichum_incanum__KZL82383.1
Aureobasidium_pullulans__THX95964.1
Aureobasidium_pullulans__THV70249.1
Aureobasidium_pullulans__THY11172.1
Aureobasidium_pullulans__THY81675.1
Aureobasidium_pullulans__THY15919.1
Aureobasidium_pullulans__THY37065.1
Aureobasidium_pullulans__THZ19739.1
Aureobasidium_subglaciale_EXF-2481__XP_013340531.1
Dothistroma_septosporum_NZE10__EME40283.1
Diaporthe_helianthi__POS72675.1
Elsinoe_australis__TKX21158.1
Monosporascus_sp._CRB-8-3__RYP86362.1
Monosporascus_sp._MG133__RYP28376.1
Monosporascus_cannonballus__RYO81974.1
Monosporascus_cannonballus__RYO91518.1
Monosporascus_ibericus__RYP03581.1
Monosporascus_sp._5C6A__RYP75199.1
Eutypa_lata_UCREL1__EMR65886.1
Xylaria_longipes__RYC65490.1
Pyricularia_oryzae_Y34__L7IKV1.1
Pyricularia_oryzae__QBZ60033.1
Pyricularia_grisea__XP_030982074.1
Pyricularia_sp._CBS_133598__TLD30473.1
Pyricularia_pennisetigena__XP_029748636.1
Gaeumannomyces_tritici_R3-111a-1__XP_009228000.1
Diaporthe_ampelina__KKY39034.1
Colletotrichum_higginsianum_IMI_349063__XP_018158055.1
Colletotrichum_higginsianum__TIC95244.1
Colletotrichum_sp_KHC_GENE_00005204-RA
Hortaea_thailandica__TKA23384.1
Botrytis_cinerea_B05.10__XP_024550392.1
Botrytis_cinerea__Q14RS2.1
Botryotinia_calthae__TEY63616.1
Botryotinia_narcissicola__TGO45745.1
Botrytis_galanthina__THV44258.1
Rutstroemia_sp._NJR-2017a_WRK4__PQE10596.1
Rutstroemia_sp._NJR-2017a_BBW__PQE10665.1
Rutstroemia_sp._NJR-2017a_BVV2__PQE16161.1
Pseudogymnoascus_sp._24MN13__OBT54585.1
Pseudogymnoascus_sp._VKM_F-4519_FW-2642__KFZ12041.1
Pseudogymnoascus_verrucosus__XP_018132374.1
Pseudogymnoascus_sp._WSF_3629__OBT40576.1
Pseudogymnoascus_sp._03VT05__OBT81682.1
Pseudogymnoascus_sp._05NY08__OBT74625.1
Pseudogymnoascus_sp._VKM_F-3557__KFX93681.1
Pseudogymnoascus_sp._VKM_F-4514_FW-929__KFY44472.1
Pseudogymnoascus_sp._VKM_F-3808__KFY03327.1
Pseudogymnoascus_sp._VKM_F-4520_FW-2644__KFZ24496.1
Pseudogymnoascus_sp._VKM_F-4518_FW-2643__KFZ00018.1
Pseudogymnoascus_sp._VKM_F-4520_FW-2644__KFZ24495.1
Glarea_lozoyensis_74030__EHL02057.1
Glarea_lozoyensis_ATCC_20868__XP_008085485.1
Parastagonospora_nodorum_SN15__XP_001793168.1
Pyrenophora_seminiperda_CCB06__RMZ73257.1
Hirsutella_minnesotensis_3608__KJZ69259.1
Alternaria_gaisen__KAB2108805.1
Alternaria_arborescens__RYN21293.1
Alternaria_sp._MG1__RII14465.1
Alternaria_sp._MG1__RII14464.1
Alternaria_tenuissima__RYN29453.1
Alternaria_alternata__XP_018379073.1
Alternaria_arborescens__XP_028508107.1
Stemphylium_lycopersici__KNG44597.1
Pyrenophora_tritici-repentis_Pt-1C-BFP__XP_001942482.1
Pyrenophora_tritici-repentis__KAA8620582.1
Pyrenophora_tritici-repentis__PZC97217.1
Parastagonospora_nodorum_SN15__XP_001802546.1
Bipolaris_maydis_ATCC_48331__XP_014077118.1
Bipolaris_victoriae_FI3__XP_014550457.1
Bipolaris_maydis_ATCC_48331__XP_014072544.1
Bipolaris_zeicola_26-R-13__XP_007718236.1
Pyrenophora_tritici-repentis__KAA8617806.1
Pyrenophora_tritici-repentis__PZD12322.1
Pyrenophora_tritici-repentis_Pt-1C-BFP__XP_001939959.1
Pyrenophora_teres_f._teres_0-1__EFQ85546.1
Pyrenophora_teres_f._teres__KAE8828272.1
Pyrenophora_seminiperda_CCB06__RMZ67979.1
Exserohilum_turcica_Et28A__XP_008028793.1
Pyrenophora_teres_f._teres__KAE8832665.1
Pyrenophora_teres_f._teres_0-1__EFQ89822.1
Lizonia_empirigonia__KAF1359822.1
Leptosphaeria_maculans_JN3__XP_003843016.1
Corynespora_cassiicola_Philippines__PSN59636.1
Exophiala_xenobiotica__XP_013319909.1
Elsinoe_australis__PSK60221.1
Elsinoe_australis__TKX18802.1
Aspergillus_costaricaensis_CBS_115574__XP_025535293.1
Aspergillus_niger__GAQ47445.1
Aspergillus_tubingensis_CBS_134.48__OJI80076.1
Aspergillus_luchuensis_CBS_106.47__OJZ85488.1
Aspergillus_piperis_CBS_112811__XP_025517274.1
Aspergillus_kawachii_IFO_4308__GAA87848.1
Aspergillus_neoniger_CBS_115656__XP_025479395.1
Aspergillus_vadensis_CBS_113365__XP_025557614.1
Aspergillus_phoenicis_ATCC_13157__RDK45894.1
Aspergillus_lacticoffeatus_CBS_101883__XP_025451354.1
Aspergillus_niger_ATCC_1015__EHA25025.1
Aspergillus_tubingensis_CBS_134.48__OJI87868.1
Aspergillus_niger__GAQ45797.1
Aspergillus_piperis_CBS_112811__XP_025512294.1
Aspergillus_kawachii_IFO_4308__GAA90263.1
Aspergillus_neoniger_CBS_115656__XP_025475370.1
Aspergillus_costaricaensis_CBS_115574__XP_025541300.1
Zymoseptoria_tritici_ST99CH_1E4__SMR59366.1
Zymoseptoria_tritici_IPO323__XP_003848975.1
Zymoseptoria_tritici_ST99CH_3D7__SMQ54927.1
Zymoseptoria_brevis__KJX96259.1
Ramularia_collo-cygni__XP_023622404.1
Cercospora_berteroae__PPJ54398.1
Cercospora_beticola__XP_023454114.1
Sphaerulina_musiva_SO2202__XP_016766109.1
Epicoccum_nigrum__OSS53141.1
Zymoseptoria_brevis__KJX95711.1
Coniochaeta_sp._2T2.1__KAB5582754.1
Coniochaeta_sp._2T2.1__KAB5585819.1
Coniochaeta_ligniaria_NRRL_30616__OIW27381.1
0.2
Sordariomycetes Glomerellales
Dothideomycetes Dothideales
Dothideomycetes Capnodiales
Sordariomycetes Diaporthales
Dothideomycetes Myriangiales
Sordariomycetes incertae-sedis
Sordariomycetes Xylariales
Sordariomycetes Magnaporthales
Sordariomycetes Diaporthales
Sordariomycetes Glomerellales
Dothideomycetes Capnodiales
Leotiomycetes Helotiales
Leotiomycetes incertae-sedis
Leotiomycetes Helotiales
Dothideomycetes Pleosporales
Sordariomycetes Hypocreales
Dothideomycetes Pleosporales
Dothideomycetes Pleosporales
Dothideomycetes incertae-sedis
Dothideomycetes Pleosporales
Eurotiomycetes Chaetothyriales
Dothideomycetes Myriangiales
Eurotiomycetes Eurotiales
Dothideomycetes Capnodiales
Dothideomycetes Capnodiales
Dothideomycetes Pleosporales
Dothideomycetes Pleosporales
Dothideomycetes Capnodiales
Sordariomycetes Coniochaetales

(E) ABA4

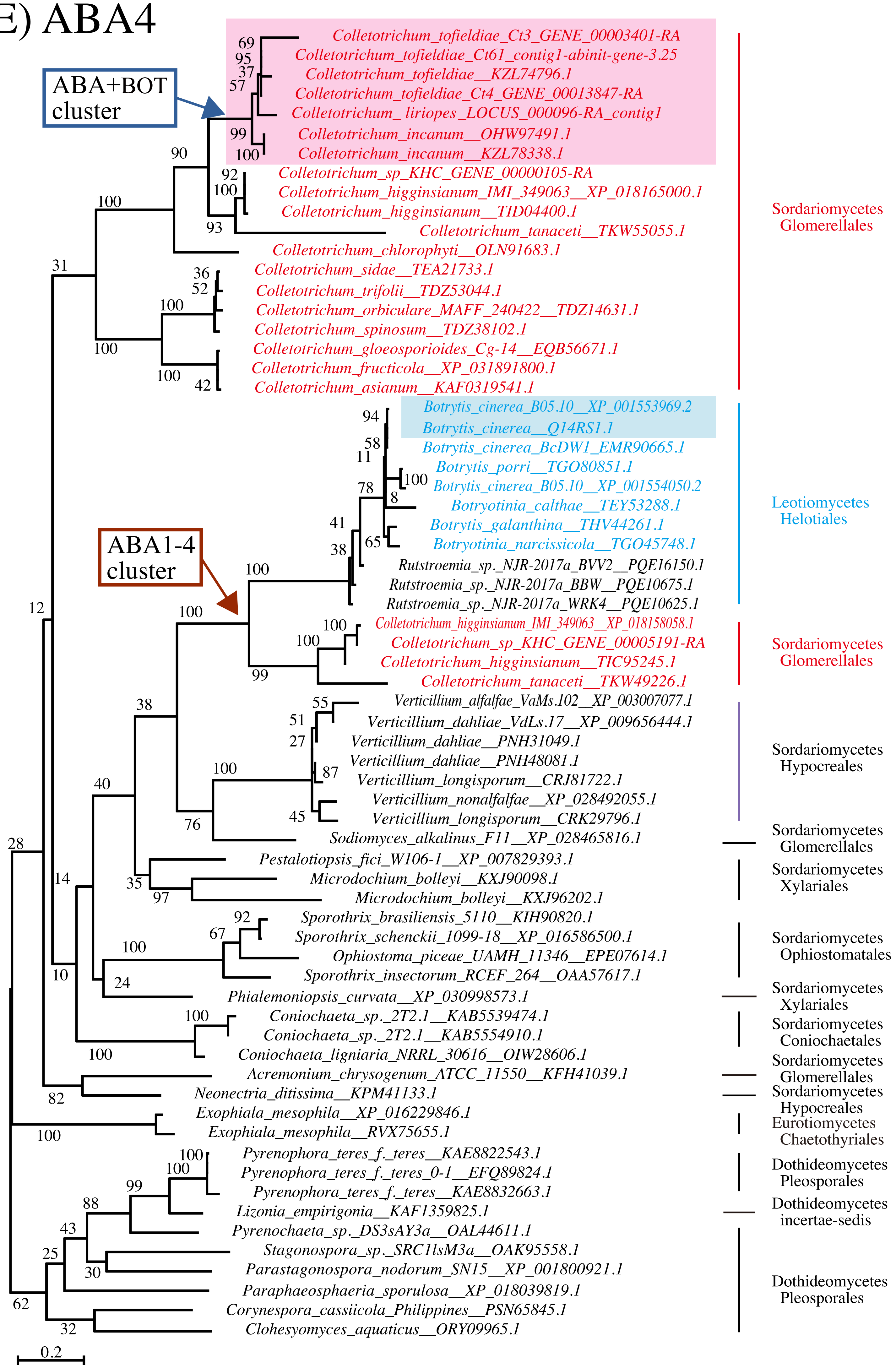
ABA+BOT cluster
ABA1-4 cluster
Colletotrichum_tofieldiae_Ct3_GENE_00003401-RA
Colletotrichum_tofieldiae_Ct61_contig1-abinit-gene-3.25
Colletotrichum_tofieldiae__KZL74796.1
Colletotrichum_tofieldiae_Ct4_GENE_00013847-RA
Colletotrichum_ liriopes_LOCUS_000096-RA_contig1
Colletotrichum_incanum__OHW97491.1
Colletotrichum_incanum__KZL78338.1
Colletotrichum_sp_KHC_GENE_00000105-RA
Colletotrichum_higginsianum_IMI_349063__XP_018165000.1
Colletotrichum_higginsianum__TID04400.1
Colletotrichum_tanaceti__TKW55055.1
Colletotrichum_chlorophyti__OLN91683.1
Colletotrichum_sidae__TEA21733.1
Colletotrichum_trifolii__TDZ53044.1
Colletotrichum_orbiculare_MAFF_240422__TDZ14631.1
Colletotrichum_spinosum__TDZ38102.1
Colletotrichum_gloeosporioides_Cg-14__EQB56671.1
Colletotrichum_fructicola__XP_031891800.1
Colletotrichum_asianum__KAF0319541.1
Sordariomycetes Glomerellales
Botrytis_cinerea_B05.10__XP_001553969.2
Botrytis_cinerea__Q14RS1.1
Botrytis_cinerea_BcDW1_EMR90665.1
Botrytis_porri__TGO80851.1
Botrytis_cinerea_B05.10__XP_001554050.2
Botryotinia_calthae__TEY53288.1
Botrytis_galanthina__THV44261.1
Botryotinia_narcissicola__TGO45748.1
Rutstroemia_sp._NJR-2017a_BVV2__PQE16150.1
Rutstroemia_sp._NJR-2017a_BBW__PQE10675.1
Rutstroemia_sp._NJR-2017a_WRK4__PQE10625.1
Leotiomycetes Helotiales
Colletotrichum_higginsianum_IMI_349063__XP_018158058.1
Colletotrichum_sp_KHC_GENE_00005191-RA
Colletotrichum_higginsianum__TIC95245.1
Colletotrichum_tanaceti__TKW49226.1
Sordariomycetes Glomerellales
Verticillium_alfalfae_VaMs.102__XP_003007077.1
Verticillium_dahliae_VdLs.17__XP_009656444.1
Verticillium_dahliae__PNH31049.1
Verticillium_dahliae__PNH48081.1
Verticillium_longisporum__CRJ81722.1
Verticillium_nonalfalfae__XP_028492055.1
Verticillium_longisporum__CRK29796.1
Sordariomycetes Hypocreales
Sodiomyces_alkalinus_F11__XP_028465816.1
Sordariomycetes Glomerellales
Pestalotiopsis_fici_W106-1__XP_007829393.1
Microdochium_bolleyi__KXJ90098.1
Microdochium_bolleyi__KXJ96202.1
Sordariomycetes Xylariales
Sporothrix_brasiliensis_5110__KIH90820.1
Sporothrix_schenckii_1099-18__XP_016586500.1
Ophiostoma_piceae_UAMH_11346__EPE07614.1
Sporothrix_insectorum_RCEF_264__OAA57617.1
Sordariomycetes Ophiostomatales
Phialemoniopsis_curvata__XP_030998573.1
Sordariomycetes Xylariales
Coniochaeta_sp._2T2.1__KAB5539474.1
Coniochaeta_sp._2T2.1__KAB5554910.1
Coniochaeta_ligniaria_NRRL_30616__OIW28606.1
Sordariomycetes Coniochaetales
Acremonium_chrysogenum_ATCC_11550__KFH41039.1
Sordariomycetes Glomerellales
Neonectria_ditissima__KPM41133.1
Sordariomycetes Hypocreales
Exophiala_mesophila__XP_016229846.1
Exophiala_mesophila__RVX75655.1
Eurotiomycetes Chaetothyriales
Pyrenophora_teres_f._teres__KAE8822543.1
Pyrenophora_teres_f._teres_0-1__EFQ89824.1
Pyrenophora_teres_f._teres__KAE8832663.1
Dothideomycetes Pleosporales
Lizonia_empirigonia__KAF1359825.1
Dothideomycetes incertae-sedis
Pyrenochaeta_sp._DS3sAY3a__OAL44611.1
Stagonospora_sp._SRC1lsM3a__OAK95558.1
Parastagonospora_nodorum_SN15__XP_001800921.1
Paraphaeosphaeria_sporulosa__XP_018039819.1
Corynespora_cassiicola_Philippines__PSN65845.1
Clohesyomyces_aquaticus__ORY09965.1
Dothideomycetes Pleosporales
0.2

(F) BOT1

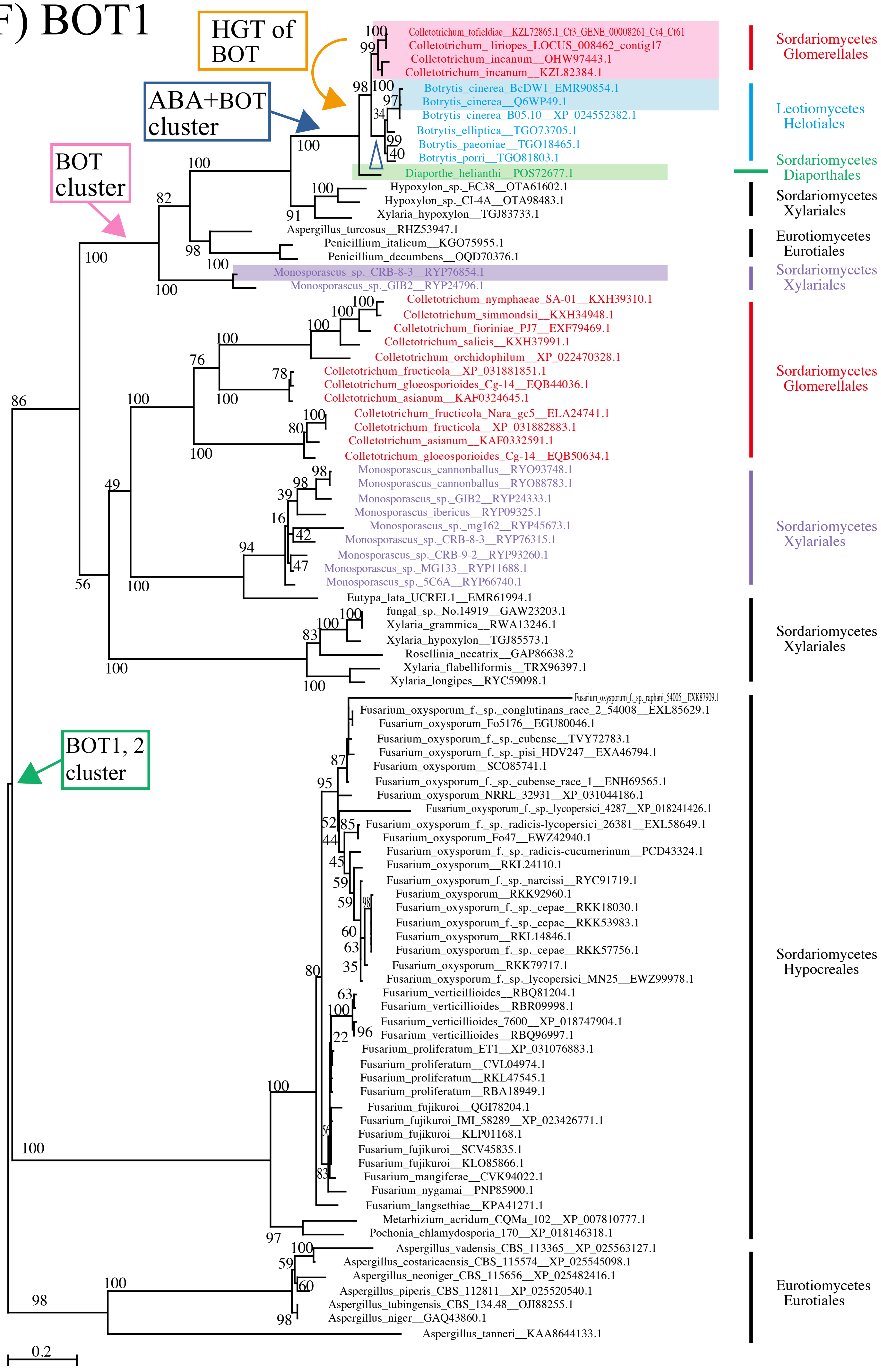

HGT of BOT
ABA+BOT cluster
BOT cluster
BOT1, 2 cluster
Colletotrichum_tofieldiae__KZL72865.1_Ct3_GENE_00008261_Ct4_Ct61
Colletotrichum_ liriopes_LOCUS_008462_contig17
Colletotrichum_incanum__OHW97443.1
Colletotrichum_incanum__KZL82384.1
Botrytis_cinerea_BcDW1_EMR90854.1
Botrytis_cinerea__Q6WP49.1
Botrytis_cinerea_B05.10__XP_024552382.1
Botrytis_elliptica__TGO73705.1
Botrytis_paeoniae__TGO18465.1
Botrytis_porri__TGO81803.1
Diaporthe_helianthi__POS72677.1
Hypoxylon_sp._EC38__OTA61602.1
Hypoxylon_sp._CI-4A__OTA98483.1
Xylaria_hypoxylon__TGJ83733.1
Aspergillus_turcosus__RHZ53947.1
Penicillium_italicum__KGO75955.1
Penicillium_decumbens__OQD70376.1
Monosporascus_sp._CRB-8-3__RYP76854.1
Monosporascus_sp._GIB2__RYP24796.1
Colletotrichum_nymphaeae_SA-01__KXH39310.1
Colletotrichum_simmondsii__KXH34948.1
Colletotrichum_fioriniae_PJ7__EXF79469.1
Colletotrichum_salicis__KXH37991.1
Colletotrichum_orchidophilum__XP_022470328.1
Colletotrichum_fructicola__XP_031881851.1
Colletotrichum_gloeosporioides_Cg-14__EQB44036.1
Colletotrichum_asianum__KAF0324645.1
Colletotrichum_fructicola_Nara_gc5__ELA24741.1
Colletotrichum_fructicola__XP_031882883.1
Colletotrichum_asianum__KAF0332591.1
Colletotrichum_gloeosporioides_Cg-14__EQB50634.1
Monosporascus_cannonballus__RYO93748.1
Monosporascus_cannonballus__RYO88783.1
Monosporascus_sp._GIB2__RYP24333.1
Monosporascus_ibericus__RYP09325.1
Monosporascus_sp._mg162__RYP45673.1
Monosporascus_sp._CRB-8-3__RYP76315.1
Monosporascus_sp._CRB-9-2__RYP93260.1
Monosporascus_sp._MG133__RYP11688.1
Monosporascus_sp._5C6A__RYP66740.1
Eutypa_lata_UCREL1__EMR61994.1
fungal_sp._No.14919__GAW23203.1
Xylaria_grammica__RWA13246.1
Xylaria_hypoxylon__TGJ85573.1
Rosellinia_necatrix__GAP86638.2
Xylaria_flabelliformis__TRX96397.1
Xylaria_longipes__RYC59098.1
Fusarium_oxysporum_f._sp._raphani_54005__EXK87909.1
Fusarium_oxysporum_f._sp._conglutinans_race_2_54008__EXL85629.1
Fusarium_oxysporum_Fo5176__EGU80046.1
Fusarium_oxysporum_f._sp._cubense__TVY72783.1
Fusarium_oxysporum_f._sp._pisi_HDV247__EXA46794.1
Fusarium_oxysporum__SCO85741.1
Fusarium_oxysporum_f._sp._cubense_race_1__ENH69565.1
Fusarium_oxysporum_NRRL_32931__XP_031044186.1
Fusarium_oxysporum_f._sp._lycopersici_4287__XP_018241426.1
Fusarium_oxysporum_f._sp._radicis-lycopersici_26381__EXL58649.1
Fusarium_oxysporum_Fo47__EWZ42940.1
Fusarium_oxysporum_f._sp._radicis-cucumerinum__PCD43324.1
Fusarium_oxysporum__RKL24110.1
Fusarium_oxysporum_f._sp._narcissi__RYC91719.1
Fusarium_oxysporum__RKK92960.1
Fusarium_oxysporum_f._sp._cepae__RKK18030.1
Fusarium_oxysporum_f._sp._cepae__RKK53983.1
Fusarium_oxysporum__RKL14846.1
Fusarium_oxysporum_f._sp._cepae__RKK57756.1
Fusarium_oxysporum__RKK79717.1
Fusarium_oxysporum_f._sp._lycopersici_MN25__EWZ99978.1
Fusarium_verticillioides__RBQ81204.1
Fusarium_verticillioides__RBR09998.1
Fusarium_verticillioides_7600__XP_018747904.1
Fusarium_verticillioides__RBQ96997.1
Fusarium_proliferatum_ET1__XP_031076883.1
Fusarium_proliferatum__CVL04974.1
Fusarium_proliferatum__RKL47545.1
Fusarium_proliferatum__RBA18949.1
Fusarium_fujikuroi__QGI78204.1
Fusarium_fujikuroi_IMI_58289__XP_023426771.1
Fusarium_fujikuroi__KLP01168.1
Fusarium_fujikuroi__SCV45835.1
Fusarium_fujikuroi__KLO85866.1
Fusarium_mangiferae__CVK94022.1
Fusarium_nygamai__PNP85900.1
Fusarium_langsethiae__KPA41271.1
Metarhizium_acridum_CQMa_102__XP_007810777.1
Pochonia_chlamydosporia_170__XP_018146318.1
Aspergillus_vadensis_CBS_113365__XP_025563127.1
Aspergillus_costaricaensis_CBS_115574__XP_025545098.1
Aspergillus_neoniger_CBS_115656__XP_025482416.1
Aspergillus_piperis_CBS_112811__XP_025520540.1
Aspergillus_tubingensis_CBS_134.48__OJI88255.1
Aspergillus_niger__GAQ43860.1
Aspergillus_tanneri__KAA8644133.1
Sordariomycetes Glomerellales
Leotiomycetes Helotiales
Sordariomycetes Diaporthales
Sordariomycetes Xylariales
Eurotiomycetes Eurotiales
Sordariomycetes Xylariales
Sordariomycetes Glomerellales
Sordariomycetes Xylariales
Sordariomycetes Xylariales
Sordariomycetes Hypocreales
Eurotiomycetes Eurotiales
0.2

(G) BOT2

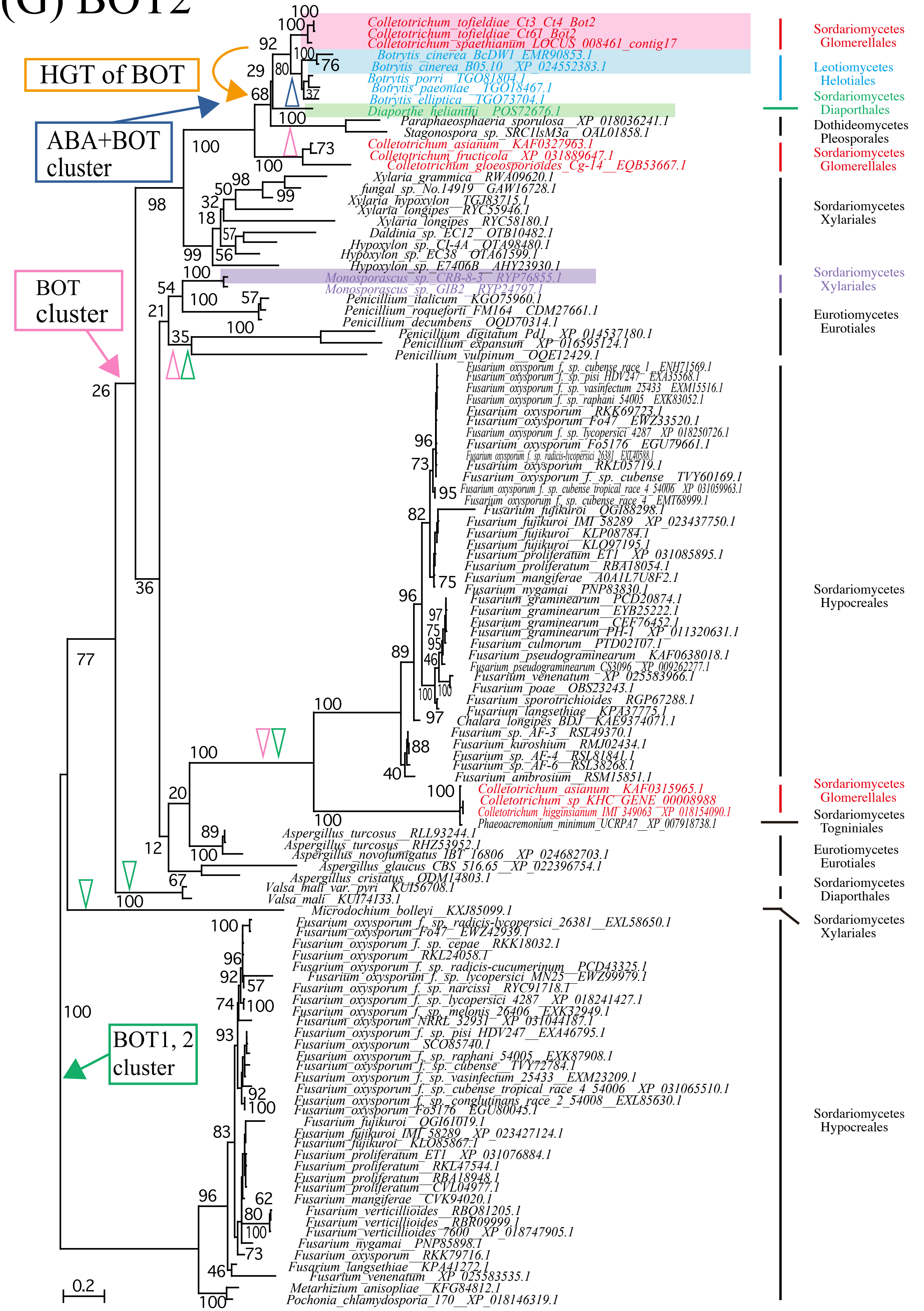
HGT of BOT
ABA+BOT cluster
BOT cluster
BOT1, 2 cluster
Colletotrichum_tofieldiae_Ct3_Ct4_Bot2
Colletotrichum_tofieldiae_Ct61_Bot2
Colletotrichum_spaethianum_LOCUS_008461_contig17
Botrytis_cinerea_BcDW1_EMR90853.1
Botrytis_cinerea_B05.10_XP_024552383.1
Botrytis_porri_TGO81804.1
Botrytis_paeoniae_TGO18467.1
Botrytis_elliptica_TGO73704.1
Diaporthe_helianthi_POS72676.1
Paraphaeosphaeria_sporulosa_XP_018036241.1
Stagonospora_sp._SRC1lsM3a_OAL01858.1
Colletotrichum_asianum_KAF0327963.1
Colletotrichum_fructicola_XP_031889647.1
Colletotrichum_gloeosporioides_Cg-14_EQB53667.1
Xylaria_grammica_RWA09620.1
fungal_sp._No.14919_GAW16728.1
Xylaria_hypoxylon_TGJ83715.1
Xylaria_longipes_RYC55946.1
Xylaria_longipes_RYC58180.1
Daldinia_sp._EC12_OTB10482.1
Hypoxylon_sp._CI-4A_OTA98480.1
Hypoxylon_sp._EC38_OTA61599.1
Hypoxylon_sp._E7406B_AHY23930.1
Monosporascus_sp._CRB-8-3_RYP76855.1
Monosporascus_sp._GIB2_RYP24797.1
Penicillium_italicum_KGO75960.1
Penicillium_roqueforti_FM164_CDM27661.1
Penicillium_decumbens_OQD70314.1
Penicillium_digitatum_Pd1_XP_014537180.1
Penicillium_expansum_XP_016595124.1
Penicillium_vulpinum_OQE12429.1
Fusarium_oxysporum_f._sp._cubense_race_1_ENH71569.1
Fusarium_oxysporum_f._sp._pisi_HDV247_EXA35568.1
Fusarium_oxysporum_f._sp._vasinfectum_25433_EXM15516.1
Fusarium_oxysporum_f._sp._raphani_54005_EXK83052.1
Fusarium_oxysporum_RKK69723.1
Fusarium_oxysporum_Fo47_EWZ33520.1
Fusarium_oxysporum_f._sp._lycopersici_4287_XP_018250726.1
Fusarium_oxysporum_Fo5176_EGU79661.1
Fusarium_oxysporum_f._sp._radicis-lycopersici_26381_EXL40588.1
Fusarium_oxysporum_RKL05719.1
Fusarium_oxysporum_f._sp._cubense_TVY60169.1
Fusarium_oxysporum_f._sp._cubense_tropical_race_4_54006_XP_031059963.1
Fusarium_oxysporum_f._sp._cubense_race_4_EMT68999.1
Fusarium_fujikuroi_OGI88298.1
Fusarium_fujikuroi_IMI_58289_XP_023437750.1
Fusarium_fujikuroi_KLP08784.1
Fusarium_fujikuroi_KLQ97195.1
Fusarium_proliferatum_ET1_XP_031085895.1
Fusarium_proliferatum_RBA18054.1
Fusarium_mangiferae_A0A1L7U8F2.1
Fusarium_nygamai_PNP83830.1
Fusarium_graminearum_PCD20874.1
Fusarium_graminearum_EYB25222.1
Fusarium_graminearum_CEF76452.1
Fusarium_graminearum_PH-1_XP_011320631.1
Fusarium_culmorum_PTD02107.1
Fusarium_pseudograminearum_KAF0638018.1
Fusarium_pseudograminearum_CS3096_XP_009262277.1
Fusarium_venenatum_XP_025583966.1
Fusarium_poae_OBS23243.1
Fusarium_sporotrichioides_RGP67288.1
Fusarium_langsethiae_KPA37775.1
Chalara_longipes_BDJ_KAE9374071.1
Fusarium_sp._AF-3_RSL49370.1
Fusarium_kuroshium_RMJ02434.1
Fusarium_sp._AF-4_RSL81841.1
Fusarium_sp._AF-6_RSL38268.1
Fusarium_ambrosium_RSM15851.1
Colletotrichum_asianum_KAF0315965.1
Colletotrichum_sp_KHC_GENE_00008988
Colletotrichum_higginsianum_IMI_349063_XP_018154090.1
Phaeoacremonium_minimum_UCRPA7_XP_007918738.1
Aspergillus_turcosus_RLL93244.1
Aspergillus_turcosus_RHZ53952.1
Aspergillus_novofumigatus_IBT_16806_XP_024682703.1
Aspergillus_glaucus_CBS_516.65_XP_022396754.1
Aspergillus_cristatus_ODM14803.1
Valsa_mali_var._pyri_KUI56708.1
Valsa_mali_KUI74133.1
Microdochium_bolleyi_KXJ85099.1
Fusarium_oxysporum_f._sp._radicis-lycopersici_26381_EXL58650.1
Fusarium_oxysporum_Fo47_EWZ42939.1
Fusarium_oxysporum_f._sp._cepae_RKK18032.1
Fusarium_oxysporum_RKL24058.1
Fusarium_oxysporum_f._sp._radicis-cucumerinum_PCD43325.1
Fusarium_oxysporum_f._sp._lycopersici_MN25_EWZ99979.1
Fusarium_oxysporum_f._sp._narcissi_RYC91718.1
Fusarium_oxysporum_f._sp._lycopersici_4287_XP_018241427.1
Fusarium_oxysporum_f._sp._melonis_26406_EXK32949.1
Fusarium_oxysporum_NRRL_32931_XP_031044187.1
Fusarium_oxysporum_f._sp._pisi_HDV247_EXA46795.1
Fusarium_oxysporum_SCO85740.1
Fusarium_oxysporum_f._sp._raphani_54005_EXK87908.1
Fusarium_oxysporum_f._sp._cubense_TVY72784.1
Fusarium_oxysporum_f._sp._vasinfectum_25433_EXM23209.1
Fusarium_oxysporum_f._sp._cubense_tropical_race_4_54006_XP_031065510.1
Fusarium_oxysporum_f._sp._conglutinans_race_2_54008_EXL85630.1
Fusarium_oxysporum_Fo5176_EGU80045.1
Fusarium_fujikuroi_OGI61019.1
Fusarium_fujikuroi_IMI_58289_XP_023427124.1
Fusarium_fujikuroi_KLO85867.1
Fusarium_proliferatum_ET1_XP_031076884.1
Fusarium_proliferatum_RKL47544.1
Fusarium_proliferatum_RBA18948.1
Fusarium_proliferatum_CVL04977.1
Fusarium_mangiferae_CVK94020.1
Fusarium_verticillioides_RBQ81205.1
Fusarium_verticillioides_RBR09999.1
Fusarium_verticillioides_7600_XP_018747905.1
Fusarium_nygamai_PNP85898.1
Fusarium_oxysporum_RKK79716.1
Fusarium_langsethiae_KPA41272.1
Fusarium_venenatum_XP_025583535.1
Metarhizium_anisopliae_KFG84812.1
Pochonia_chlamydosporia_170_XP_018146319.1
Sordariomycetes Glomerellales
Leotiomycetes Helotiales
Sordariomycetes Diaporthales
Dothideomycetes Pleosporales
Sordariomycetes Glomerellales
Sordariomycetes Xylariales
Sordariomycetes Xylariales
Eurotiomycetes Eurotiales
Sordariomycetes Hypocreales
Sordariomycetes Glomerellales
Sordariomycetes Togniniales
Eurotiomycetes Eurotiales
Sordariomycetes Diaporthales
Sordariomycetes Xylariales
Sordariomycetes Hypocreales
0.2

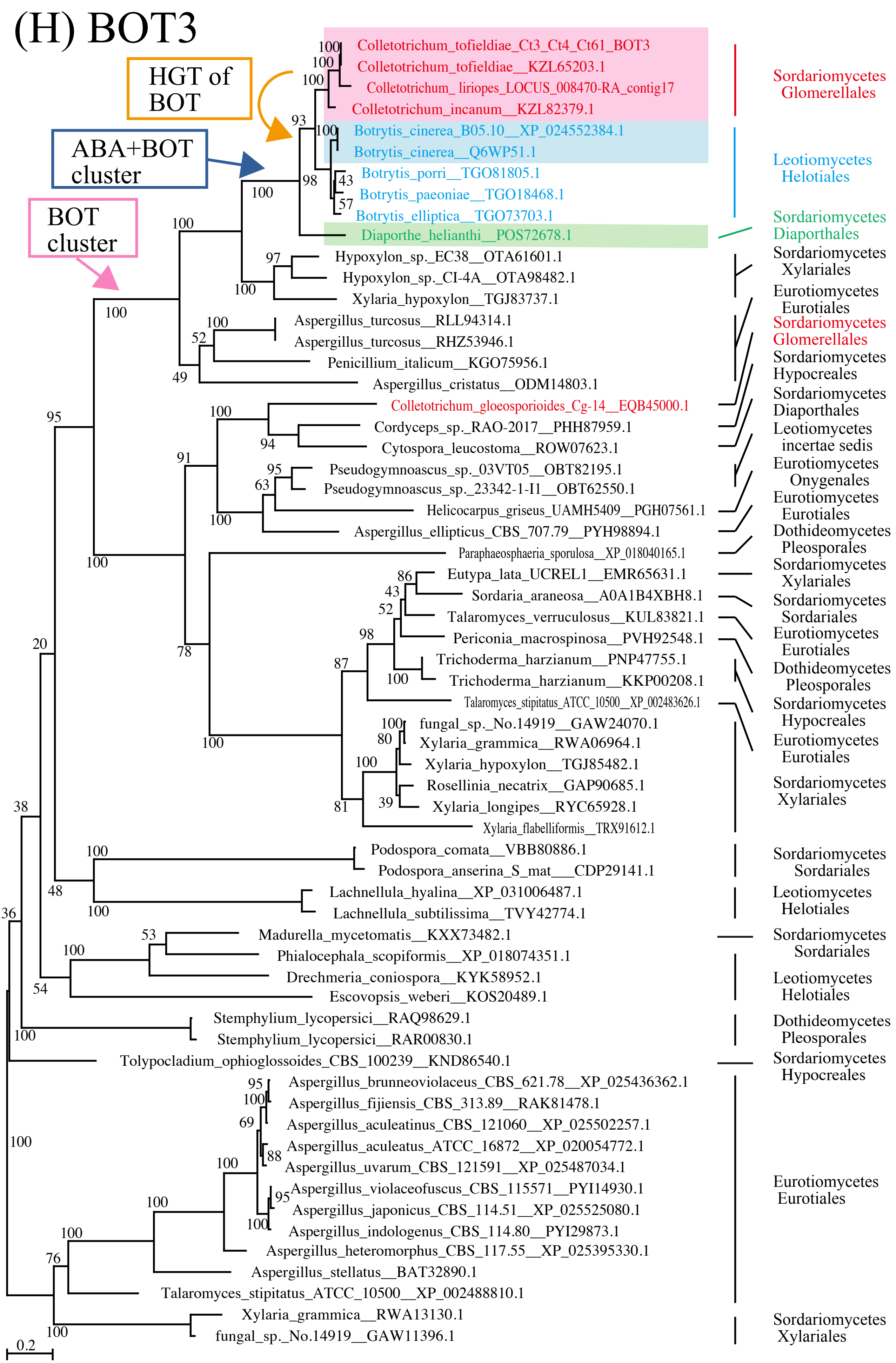
(H) BOT3
HGT of BOT
ABA+BOT cluster
BOT cluster
Colletotrichum_tofieldiae_Ct3_Ct4_Ct61_BOT3
Colletotrichum_tofieldiae__KZL65203.1
Colletotrichum_ liriopes_LOCUS_008470-RA_contig17
Colletotrichum_incanum__KZL82379.1
Botrytis_cinerea_B05.10__XP_024552384.1
Botrytis_cinerea__Q6WP51.1
Botrytis_porri__TGO81805.1
Botrytis_paeoniae__TGO18468.1
Botrytis_elliptica__TGO73703.1
Diaporthe_helianthi__POS72678.1
Hypoxylon_sp._EC38__OTA61601.1
Hypoxylon_sp._CI-4A__OTA98482.1
Xylaria_hypoxylon__TGJ83737.1
Aspergillus_turcosus__RLL94314.1
Aspergillus_turcosus__RHZ53946.1
Penicillium_italicum__KGO75956.1
Aspergillus_cristatus__ODM14803.1
Colletotrichum_gloeosporioides_Cg-14__EQB45000.1
Cordyceps_sp._RAO-2017__PHH87959.1
Cytospora_leucostoma__ROW07623.1
Pseudogymnoascus_sp._03VT05__OBT82195.1
Pseudogymnoascus_sp._23342-1-I1__OBT62550.1
Helicocarpus_griseus_UAMH5409__PGH07561.1
Aspergillus_ellipticus_CBS_707.79__PYH98894.1
Paraphaeosphaeria_sporulosa__XP_018040165.1
Eutypa_lata_UCREL1__EMR65631.1
Sordaria_araneosa__A0A1B4XBH8.1
Talaromyces_verruculosus__KUL83821.1
Periconia_macrospinosa__PVH92548.1
Trichoderma_harzianum__PNP47755.1
Trichoderma_harzianum__KKP00208.1
Talaromyces_stipitatus_ATCC_10500__XP_002483626.1
fungal_sp._No.14919__GAW24070.1
Xylaria_grammica__RWA06964.1
Xylaria_hypoxylon__TGJ85482.1
Rosellinia_necatrix__GAP90685.1
Xylaria_longipes__RYC65928.1
Xylaria_flabelliformis__TRX91612.1
Podospora_comata__VBB80886.1
Podospora_anserina_S_mat___CDP29141.1
Lachnellula_hyalina__XP_031006487.1
Lachnellula_subtilissima__TVY42774.1
Madurella_mycetomatis__KXX73482.1
Phialocephala_scopiformis__XP_018074351.1
Drechmeria_coniospora__KYK58952.1
Escovopsis_weberi__KOS20489.1
Stemphylium_lycopersici__RAQ98629.1
Stemphylium_lycopersici__RAR00830.1
Tolypocladium_ophioglossoides_CBS_100239__KND86540.1
Aspergillus_brunneoviolaceus_CBS_621.78__XP_025436362.1
Aspergillus_fijiensis_CBS_313.89__RAK81478.1
Aspergillus_aculeatinus_CBS_121060__XP_025502257.1
Aspergillus_aculeatus_ATCC_16872__XP_020054772.1
Aspergillus_uvarum_CBS_121591__XP_025487034.1
Aspergillus_violaceofuscus_CBS_115571__PYI14930.1
Aspergillus_japonicus_CBS_114.51__XP_025525080.1
Aspergillus_indologenus_CBS_114.80__PYI29873.1
Aspergillus_heteromorphus_CBS_117.55__XP_025395330.1
Aspergillus_stellatus__BAT32890.1
Talaromyces_stipitatus_ATCC_10500__XP_002488810.1
Xylaria_grammica__RWA13130.1
fungal_sp._No.14919__GAW11396.1
0.2
Sordariomycetes Glomerellales
Leotiomycetes Helotiales
Sordariomycetes Diaporthales
Sordariomycetes Xylariales
Eurotiomycetes Eurotiales
Sordariomycetes Glomerellales
Sordariomycetes Hypocreales
Sordariomycetes Diaporthales
Leotiomycetes incertae sedis
Eurotiomycetes Onygenales
Eurotiomycetes Eurotiales
Dothideomycetes Pleosporales
Sordariomycetes Xylariales
Sordariomycetes Sordariales
Eurotiomycetes Eurotiales
Dothideomycetes Pleosporales
Sordariomycetes Hypocreales
Eurotiomycetes Eurotiales
Sordariomycetes Xylariales
Sordariomycetes Sordariales
Leotiomycetes Helotiales
Sordariomycetes Sordariales
Leotiomycetes Helotiales
Dothideomycetes Pleosporales
Sordariomycetes Hypocreales
Eurotiomycetes Eurotiales
Sordariomycetes Xylariales

(I) BOT4

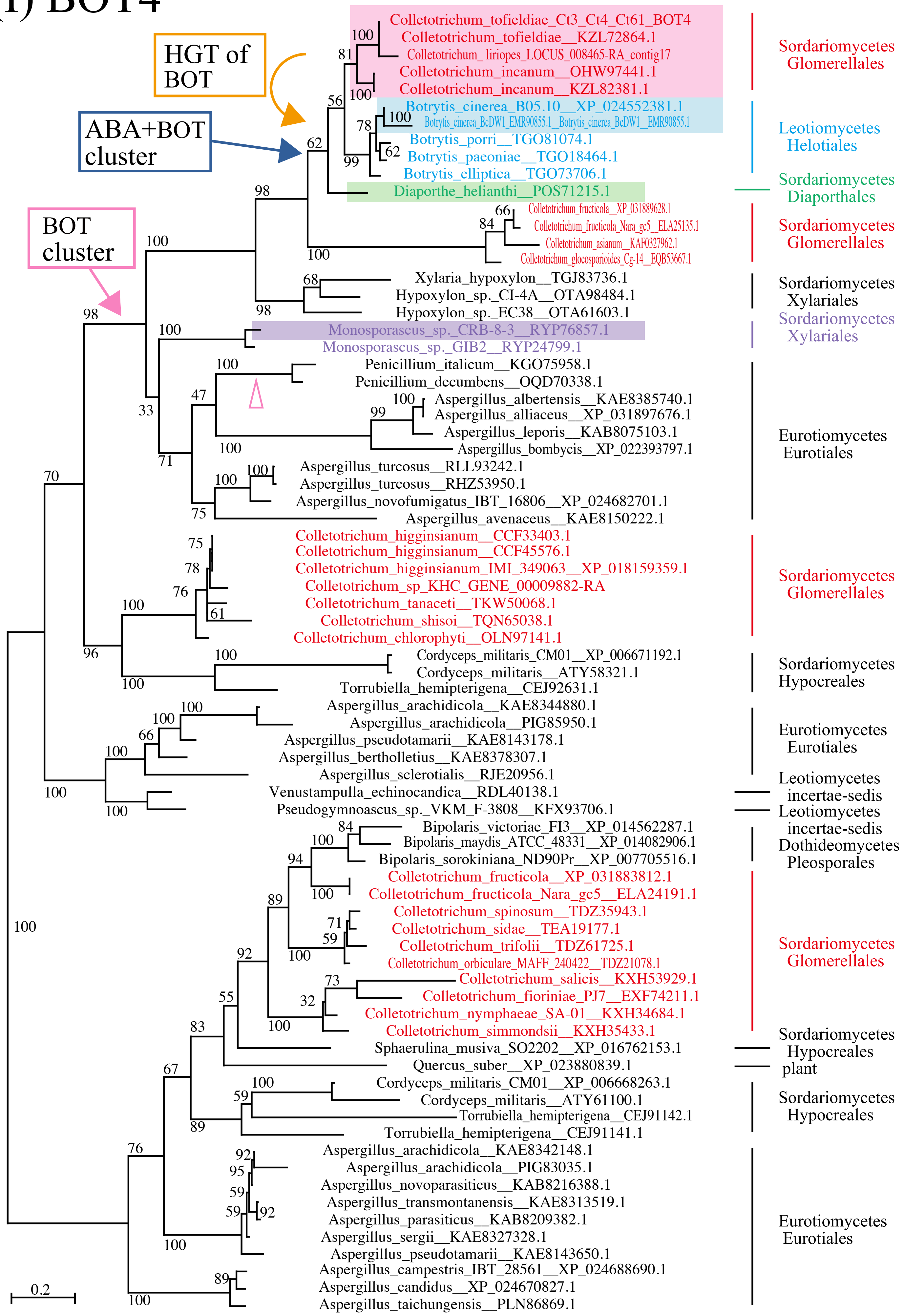

HGT of BOT
ABA+BOT cluster
BOT cluster
Colletotrichum_tofieldiae_Ct3_Ct4_Ct61_BOT4
Colletotrichum_tofieldiae__KZL72864.1
Colletotrichum_ liriopes_LOCUS_008465-RA_contig17
Colletotrichum_incanum__OHW97441.1
Colletotrichum_incanum__KZL82381.1
Botrytis_cinerea_B05.10__XP_024552381.1
Botrytis_cinerea_BcDW1_EMR90855.1__Botrytis_cinerea_BcDW1__EMR90855.1
Botrytis_porri__TGO81074.1
Botrytis_paeoniae__TGO18464.1
Botrytis_elliptica__TGO73706.1
Diaporthe_helianthi__POS71215.1
Colletotrichum_fructicola__XP_031889628.1
Colletotrichum_fructicola_Nara_gc5__ELA25135.1
Colletotrichum_asianum__KAF0327962.1
Colletotrichum_gloeosporioides_Cg-14__EQB53667.1
Xylaria_hypoxylon__TGJ83736.1
Hypoxylon_sp._CI-4A__OTA98484.1
Hypoxylon_sp._EC38__OTA61603.1
Monosporascus_sp._CRB-8-3__RYP76857.1
Monosporascus_sp._GIB2__RYP24799.1
Penicillium_italicum__KGO75958.1
Penicillium_decumbens__OQD70338.1
Aspergillus_albertensis__KAE8385740.1
Aspergillus_alliaceus__XP_031897676.1
Aspergillus_leporis__KAB8075103.1
Aspergillus_bombycis__XP_022393797.1
Aspergillus_turcosus__RLL93242.1
Aspergillus_turcosus__RHZ53950.1
Aspergillus_novofumigatus_IBT_16806__XP_024682701.1
Aspergillus_avenaceus__KAE8150222.1
Colletotrichum_higginsianum__CCF33403.1
Colletotrichum_higginsianum__CCF45576.1
Colletotrichum_higginsianum_IMI_349063__XP_018159359.1
Colletotrichum_sp_KHC_GENE_00009882-RA
Colletotrichum_tanaceti__TKW50068.1
Colletotrichum_shisoi__TQN65038.1
Colletotrichum_chlorophyti__OLN97141.1
Cordyceps_militaris_CM01__XP_006671192.1
Cordyceps_militaris__ATY58321.1
Torrubiella_hemipterigena__CEJ92631.1
Aspergillus_arachidicola__KAE8344880.1
Aspergillus_arachidicola__PIG85950.1
Aspergillus_pseudotamarii__KAE8143178.1
Aspergillus_bertholletius__KAE8378307.1
Aspergillus_sclerotialis__RJE20956.1
Venustampulla_echinocandica__RDL40138.1
Pseudogymnoascus_sp._VKM_F-3808__KFX93706.1
Bipolaris_victoriae_FI3__XP_014562287.1
Bipolaris_maydis_ATCC_48331__XP_014082906.1
Bipolaris_sorokiniana_ND90Pr__XP_007705516.1
Colletotrichum_fructicola__XP_031883812.1
Colletotrichum_fructicola_Nara_gc5__ELA24191.1
Colletotrichum_spinosum__TDZ35943.1
Colletotrichum_sidae__TEA19177.1
Colletotrichum_trifolii__TDZ61725.1
Colletotrichum_orbiculare_MAFF_240422__TDZ21078.1
Colletotrichum_salicis__KXH53929.1
Colletotrichum_fioriniae_PJ7__EXF74211.1
Colletotrichum_nymphaeae_SA-01__KXH34684.1
Colletotrichum_simmondsii__KXH35433.1
Sphaerulina_musiva_SO2202__XP_016762153.1
Quercus_suber__XP_023880839.1
Cordyceps_militaris_CM01__XP_006668263.1
Cordyceps_militaris__ATY61100.1
Torrubiella_hemipterigena__CEJ91142.1
Torrubiella_hemipterigena__CEJ91141.1
Aspergillus_arachidicola__KAE8342148.1
Aspergillus_arachidicola__PIG83035.1
Aspergillus_novoparasiticus__KAB8216388.1
Aspergillus_transmontanensis__KAE8313519.1
Aspergillus_parasiticus__KAB8209382.1
Aspergillus_sergii__KAE8327328.1
Aspergillus_pseudotamarii__KAE8143650.1
Aspergillus_campestris_IBT_28561__XP_024688690.1
Aspergillus_candidus__XP_024670827.1
Aspergillus_taichungensis__PLN86869.1
0.2
Sordariomycetes Glomerellales
Leotiomycetes Helotiales
Sordariomycetes Diaporthales
Sordariomycetes Glomerellales
Sordariomycetes Xylariales
Sordariomycetes Xylariales
Eurotiomycetes Eurotiales
Sordariomycetes Glomerellales
Sordariomycetes Hypocreales
Eurotiomycetes Eurotiales
Leotiomycetes incertae-sedis
Leotiomycetes incertae-sedis
Dothideomycetes Pleosporales
Sordariomycetes Glomerellales
Sordariomycetes Hypocreales
plant
Sordariomycetes Hypocreales
Eurotiomycetes Eurotiales

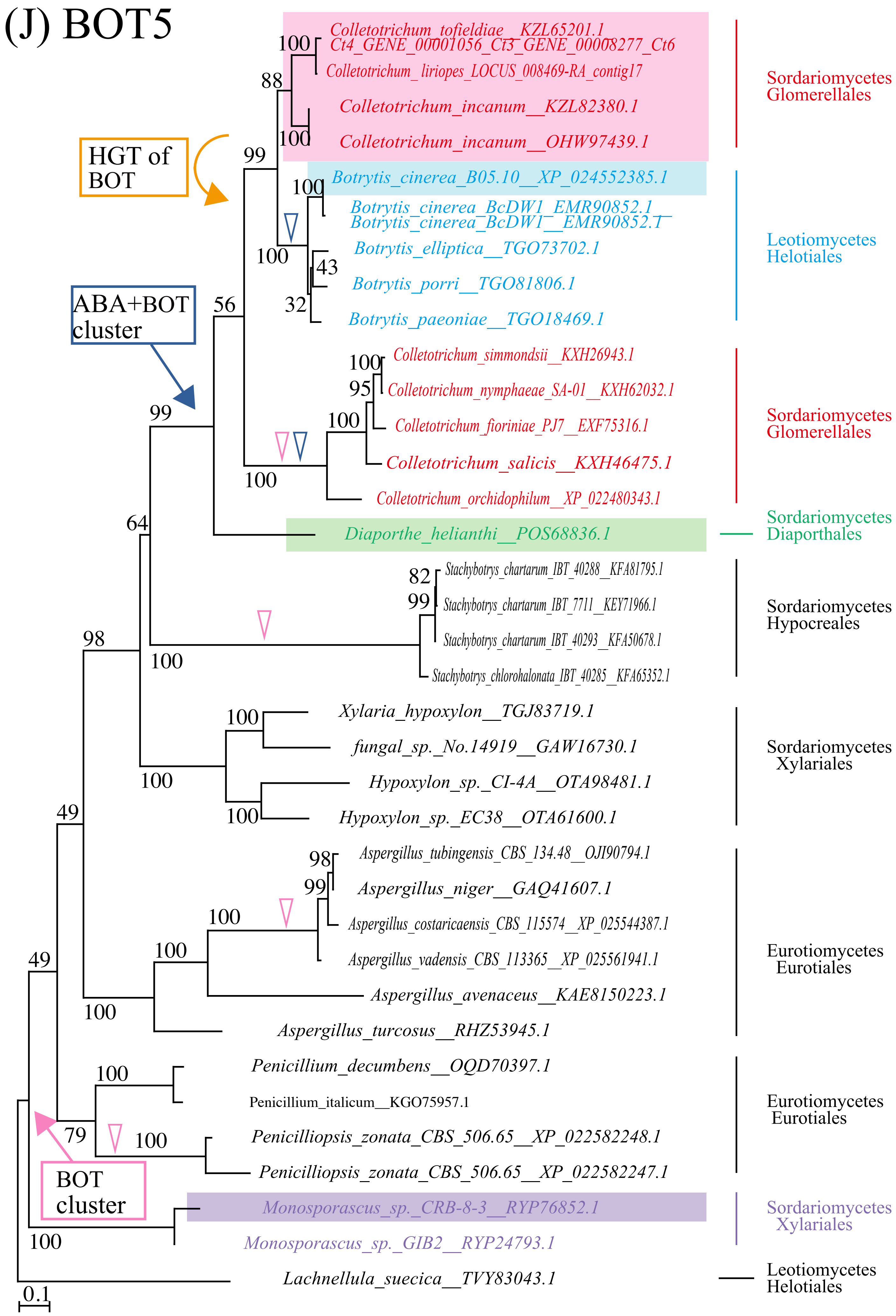
(J) BOT5
HGT of BOT
ABA+BOT cluster
BOT cluster
Colletotrichum_tofieldiae__KZL65201.1
Ct4_GENE_00001056_Ct3_GENE_00008277_Ct6
Colletotrichum_liriopes_LOCUS_008469-RA_contig17
Colletotrichum_incanum__KZL82380.1
Colletotrichum_incanum__OHW97439.1
Botrytis_cinerea_B05.10__XP_024552385.1
Botrytis_cinerea_BcDW1_EMR90852.1
Botrytis_cinerea_BcDW1__EMR90852.1
Botrytis_elliptica__TGO73702.1
Botrytis_porri__TGO81806.1
Botrytis_paeoniae__TGO18469.1
Colletotrichum_simmondsii__KXH26943.1
Colletotrichum_nymphaeae_SA-01__KXH62032.1
Colletotrichum_fioriniae_PJ7__EXF75316.1
Colletotrichum_salicis__KXH46475.1
Colletotrichum_orchidophilum__XP_022480343.1
Diaporthe_helianthi__POS68836.1
Stachybotrys_chartarum_IBT_40288__KFA81795.1
Stachybotrys_chartarum_IBT_7711__KEY71966.1
Stachybotrys_chartarum_IBT_40293__KFA50678.1
Stachybotrys_chlorohalonata_IBT_40285__KFA65352.1
Xylaria_hypoxylon__TGJ83719.1
fungal_sp._No.14919__GAW16730.1
Hypoxylon_sp._CI-4A__OTA98481.1
Hypoxylon_sp._EC38__OTA61600.1
Aspergillus_tubingensis_CBS_134.48__OJI90794.1
Aspergillus_niger__GAQ41607.1
Aspergillus_costaricaensis_CBS_115574__XP_025544387.1
Aspergillus_vadensis_CBS_113365__XP_025561941.1
Aspergillus_avenaceus__KAE8150223.1
Aspergillus_turcosus__RHZ53945.1
Penicillium_decumbens__OQD70397.1
Penicillium_italicum__KGO75957.1
Penicilliopsis_zonata_CBS_506.65__XP_022582248.1
Penicilliopsis_zonata_CBS_506.65__XP_022582247.1
Monosporascus_sp._CRB-8-3__RYP76852.1
Monosporascus_sp._GIB2__RYP24793.1
Lachnellula_suecica__TVY83043.1
Sordariomycetes Glomerellales
Leotiomycetes Helotiales
Sordariomycetes Glomerellales
Sordariomycetes Diaporthales
Sordariomycetes Hypocreales
Sordariomycetes Xylariales
Eurotiomycetes Eurotiales
Eurotiomycetes Eurotiales
Sordariomycetes Xylariales
Leotiomycetes Helotiales
100
88
100
99
100
100
43
32
56
100
95
100
99
100
64
82
99
98
100
100
100
100
49
98
99
100
100
49
100
100
79
100
100
0.1

(K) BOT6 (Zn_clus)

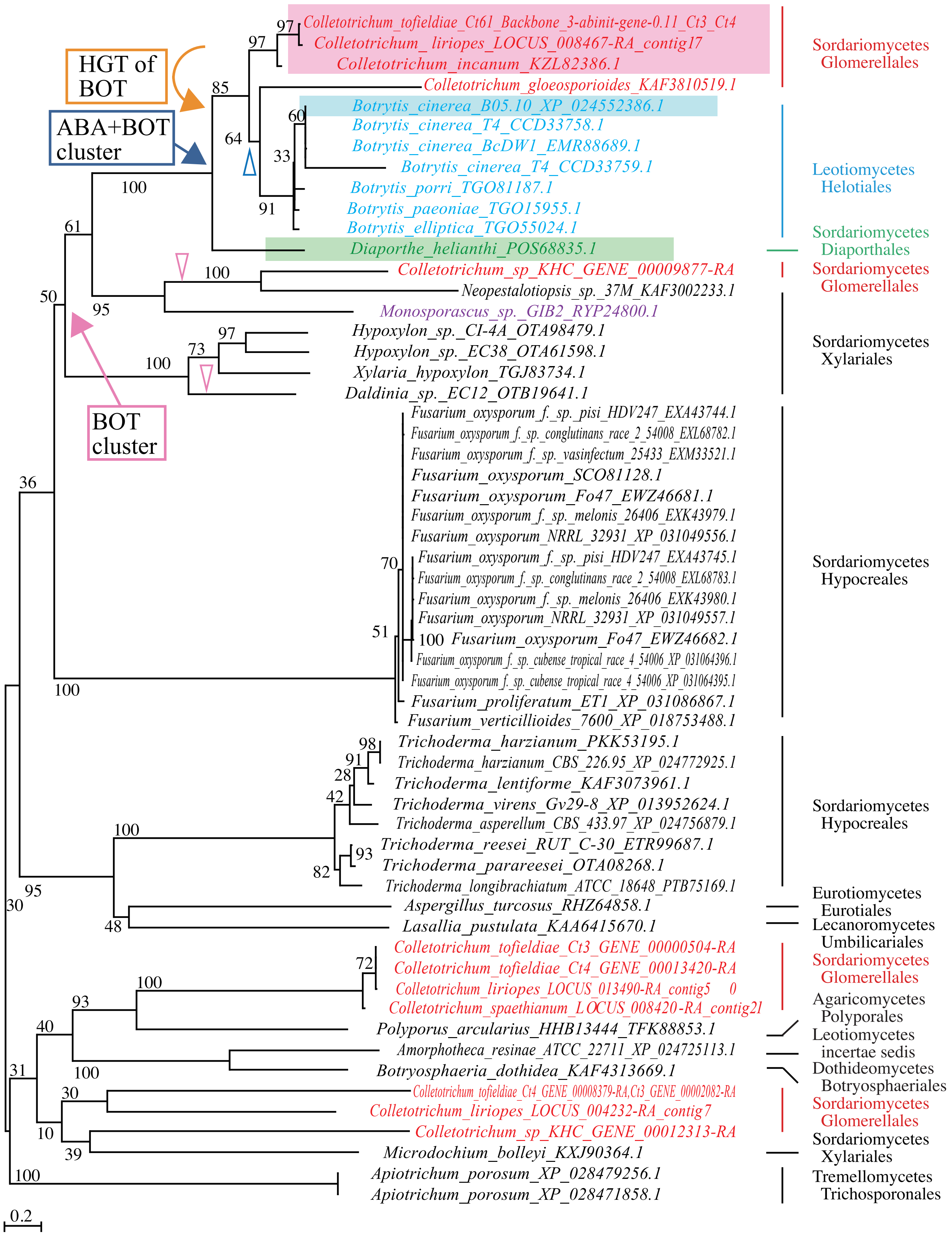

(L) BOT7 (Adh_short)

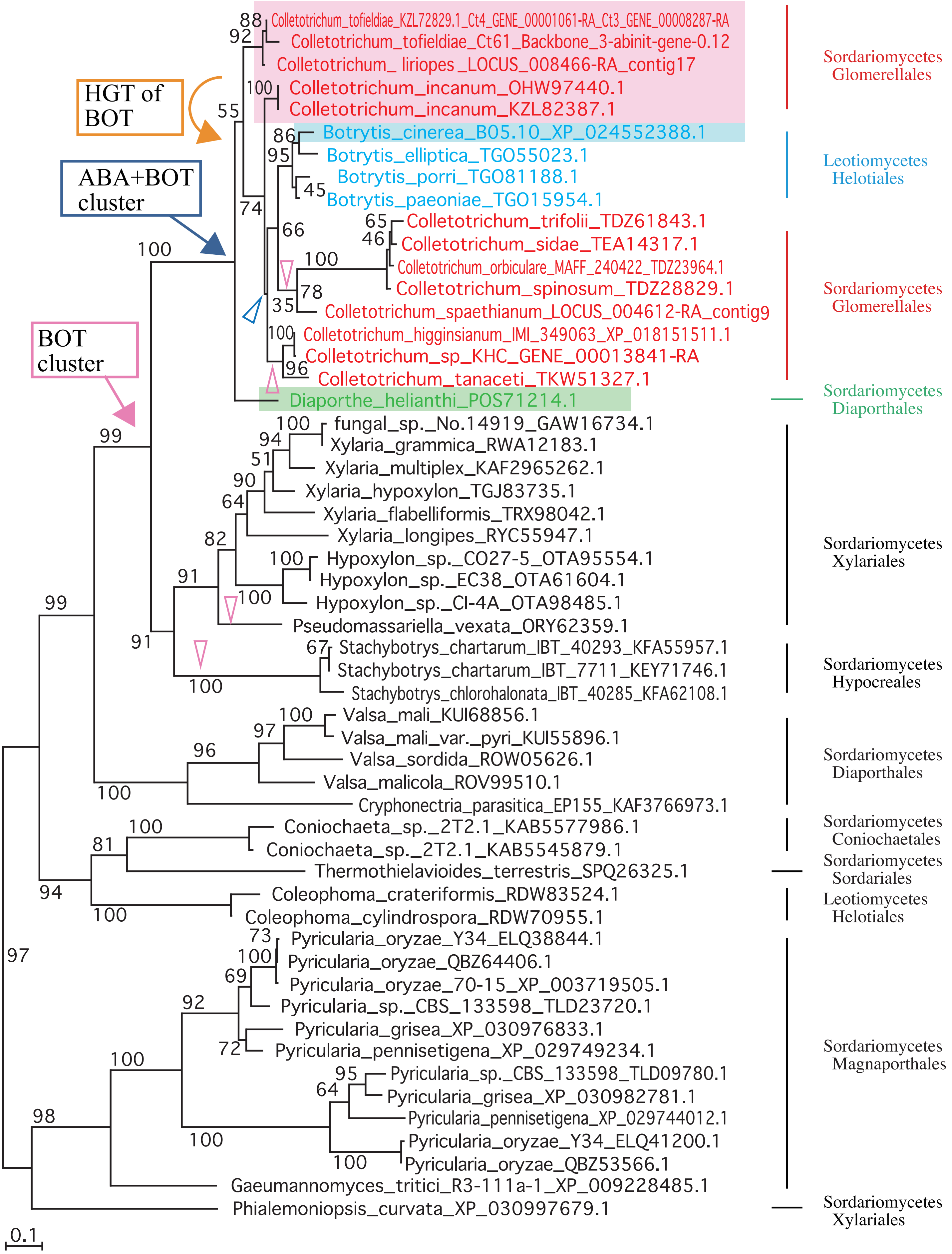

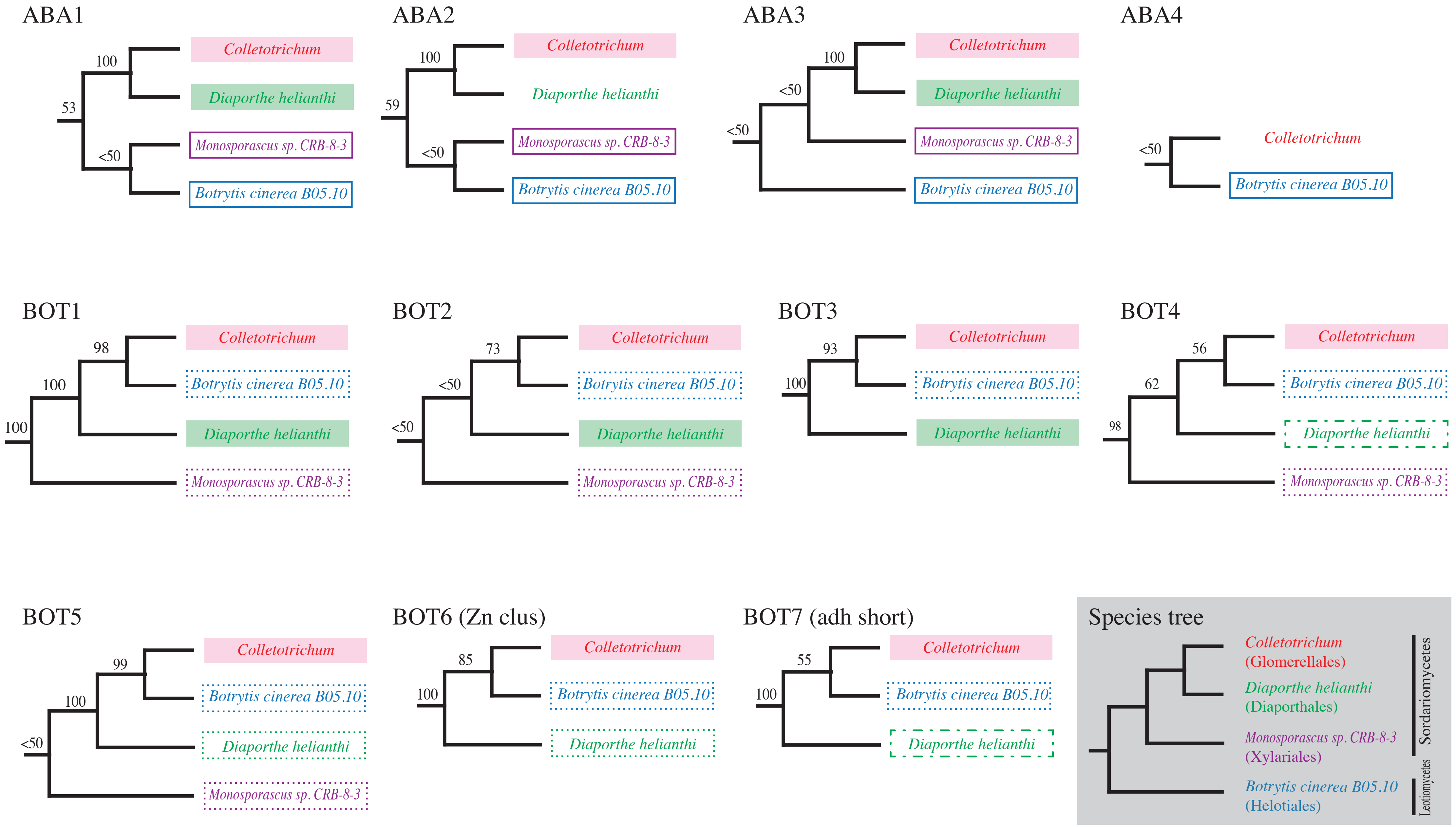

m. Phylogenetic relationship of ABA and BOT genes in the four organisms that have both ABA and BOT clusters in their genome. The relationship and branch support are based on Supplementary Figure 4b-l. The bootstrap probability is shown on the branches. The species tree of the four organisms is shown in the rectangle with a gray background. Organisms that have ABA-BOT cluster are labeled with red or green backgrounds. Organism names of each gene that is on the same molecule (contig), but not on ABA-BOT cluster, are boxed with the same solid or dotted line.

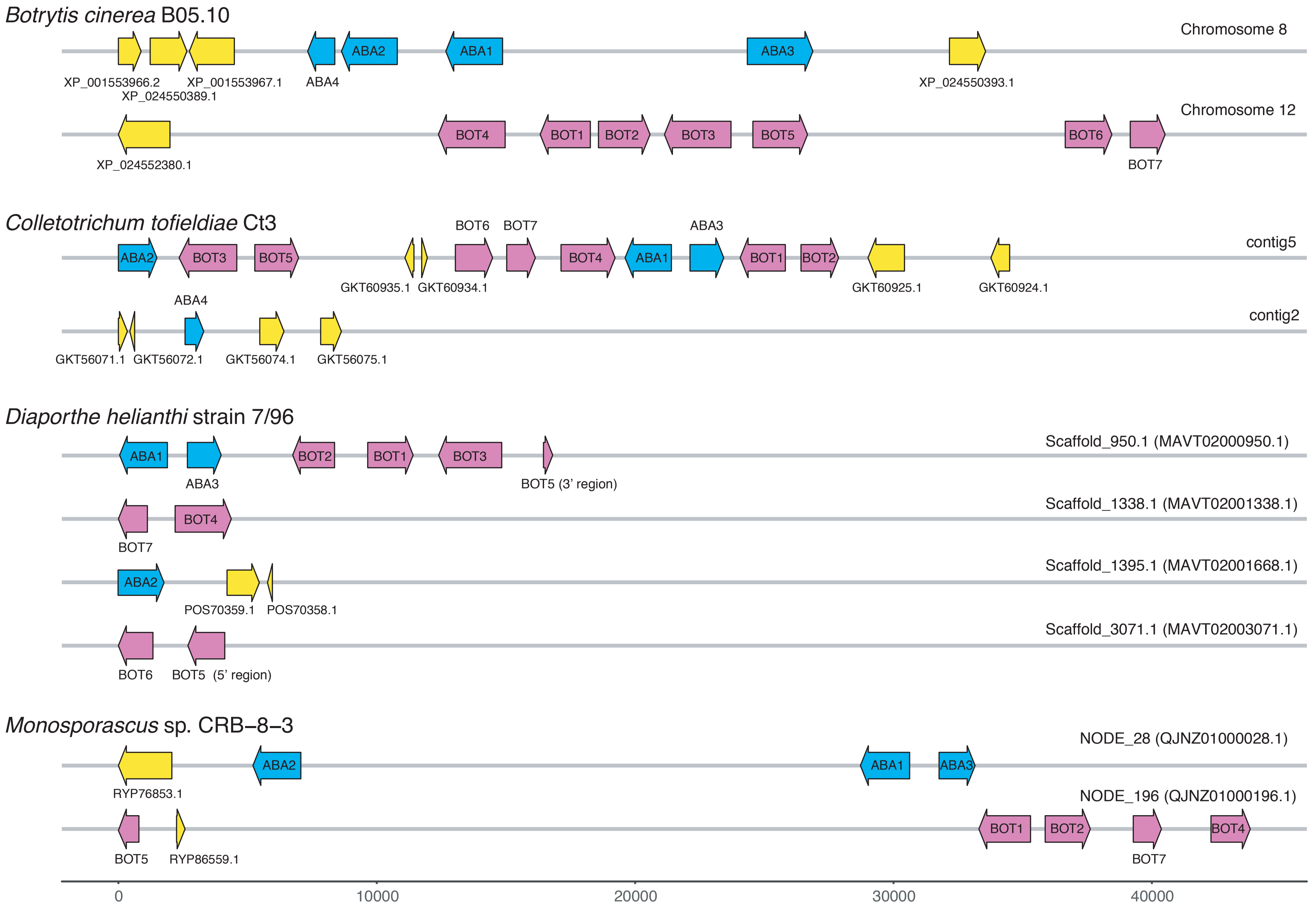

n. Schematic drawing of genetic structures of ABA and BOT gene clusters. Genes on the same molecule (chromosome or scaffold) are drawn on a line side by side. Color coding; blue: ABA genes, red: BOT genes, yellow: other genes. Bar indicates base pairs.

Supplementary Figure 4o: Amino acids alignments of Ct ABA and BOT genes.

```
CLUSTAL format alignment by MAFFT (v7.511)

ABA1
   Ct3        MSSFLITASYTSLLGAGAGILVFCYLLSALYSWNRLGAVPAASWTAHFSYLWLAKTTYSG
   Ct4        MSSFLITASYTSLLGAGAGILVFCYLLSALYSWNRLGAVPAASWTAHFSYLWLAKTTYSG
   Ct61       MSSFLITASYTSLLGAGAGILVFCYLLSALYSWNRLGAVPAASWTAHFSYLWLAKTTYSG
              ************************************************************

   Ct3        RQYWVHRSLHKDKGPLVRVGPNEVVTNDPKLVRKISGTDERWARDPFYITGKFNPYHDNM
   Ct4        RQYWVHRSLHKDKGPLVRVGPNEVVTNDPKLVRKISGTDERWARDPFYITGKFNPYHDNM
   Ct61       RQYWVHRSLHKDKGPLVRVGPNEVVTNDPKLVRKISGTDERWARDPFYITGKFNPYHDNM
              ************************************************************

   Ct3        FSILNPREHQLAKSRRLAAYSGRETPDLEIGMDTLVKTLIDMIETRYTSPVQDSRVPPLL
   Ct4        FSILNPREHQLAKSRRLAAYSGRETPDLEIGMDTLVKTLIDMIETRYTSPVQDSRVPPLL
   Ct61       FSILNPREHQLAKSRRLAAYSGRETPDLEIGMDTLVKTLIDMIETRYTSPVQDSRVPPLL
              ************************************************************

   Ct3        DLGKTSCYFTLDVITRLAFGKEVGYLDSEKDHFGFLGSLHDLWPQMSTCADIPMLRKFLF
   Ct4        DLGKTSCYFTLDVITRLAFGKEVGYLDSEKDHFGFLGSLHDLWPQMSTCADIPMLRKFLF
   Ct61       DLGKTSCYFTLDVITRLAFGKEVGYLDSEKDHFGFLGSLHDLWPQMSTCADIPMLRKFLF
              ************************************************************

   Ct3        SSFFLKFMGPKTTDKVGFGALMGVANHYVGNRFASTEKPQQDMLGSMIKHGLDQVECETE
   Ct4        SSFFLKFMGPKTTDKVGFGALMGVANHYVGNRFASTEKPQQDMLGSMIKHGLDQVECETE
   Ct61       SSFFLKFMGPKTTDKVGFGALMGVANHYVGNRFASTEKPQQDMLGSMIKHGLDQVECETE
              ************************************************************

   Ct3        GLFMIIAGTESTASAIRSALVHAMTSPLVYQKLKDEIHTAVCEGKVSSPITFQEAKALPY
   Ct4        GLFMIIAGTESTASAIRSALVHAMTSPLVYQKLKDEIHTAVCEGKVSSPITFQEAKALPY
   Ct61       GLFMIIAGTESTASAIRSALVHAMTSPLVYQKLKDEIHTAVREGKVSSPITFQEAKALPY
              ***************************************** ******************

   Ct3        LQAVIYESIRMRPPLIGYFPKVVPPGGETLLGYHLPAGTSIGTNMSAILSSTDIFGPDAG
   Ct4        LQAVIYESIRMRPPLIGYFPKVVPPGGETLLGYHLPAGTSIGTNMSAILSSTDIFGPDAG
   Ct61       LQAVIYESIRMRPPLIGYFPKVVPPGGETLLGYHLPAGTSIGTNMSAILSSTDIFGPDAG
              ************************************************************

   Ct3        VFRPERFTELDEDSCKRMKRDVELAFGSGQWMCMGKTIAFMEINKSVFEIFRAFDIQLVE
   Ct4        VFRPERFTELDEDSCKRMKRDVELAFGSGQWMCMGKTIAFMEINKSVFEIFRAFDIQLVE
   Ct61       VFRPERFTELDEDSCKRMKRDVELAFGSGQWMCMGKTIAFMEINKSVFEIFRAFDIQLVE
              ************************************************************

   Ct3        PFAPSTIATYGTFLESQMMAKVSKSDVS
   Ct4        PFAPSTIATYGTFLESQMMAKVSKSDVS
   Ct61       PFAPSTIATYGTFLESQMMAKVSKSDVS
              ****************************

ABA2
   Ct3        MDTIVTSLAKSHSLLRDAKDALPKDSLLWVTSAVTVTFTLVILARIIKARFIHPLRKFPG
   Ct4        MDTIVTSLAKSHSLLRDAKDALPKDSLLWVTSAVTVTFTLVILARIIKARFIHPLRKFPG
   Ct61       MDTIVTSLAKSHSLLRDAKDALPKDSLLWVTSAVTVTFTLVILARIIKARFIHPLRKFPG
              ************************************************************

   Ct3        PFLNSVSSLPAAYLVYRGNQPQSFKKLHEKYGPVVRTGPNELSFIGADAWEDIYGIQKVG
   Ct4        PFLNSVSSLPAAYLVYRGNQPQSFKKLHEKYGPVVRTGPNELSFIGADAWEDIYGIQKVG
   Ct61       PFLNSVSSLPAAYLVYRGNQPQSFKKLHEKYGPVVRTGPNELSFIGADAWEDIYGIQKVG
              ************************************************************

   Ct3        LNFQKDPSWLAVVSPKDGQTGLSLAPPETHTRQRNALGATFLNEALLSQEHIIQGHVDKF
   Ct4        LNFQKDPSWLAVVSPKDGQTGLSLAPPETHTRQRNALGATFLNEALLSQEHIIQGHVDKF
   Ct61       LNFQKDPSWLAVVSPKDGQTGLSLAPPETHTRQRNALGATFLNEALLSQEHIIQGHVDKF
              ************************************************************

   Ct3        MEVIKRHIAENKAIDLSHWYGYATFDVIGDVIFAEPFGCLDESEETQWMRAINDIFKSGA
   Ct4        MEVIKRHIAENKAIDLSHWYGYATFDVIGDVIFAEPFGCLDESEETQWMRAINDIFKSGA
```

```
Ct61      MEVIKRHIAENKAIDLSHWYGYATFDVIGDVIFAEPFGCLDESEETQWMRAINDIFKSGA
          ************************************************************

Ct3       WEQAFGQLAGVGTLLHKLMVKFLIPNELKSWRLKHLSKATEKTRRRMATPNPAHPDMVAH
Ct4       WEQAFGQLAGVGTLLHKLMVKFLIPNELKSWRLKHLSKATEKTRRRMATPNPAHPDMVAH
Ct61      WEQAFGQLAGVGTLLHKLMVKFLIPNELKSWRLKHLSKATEKTRRRMATPNPAHPDMVAH
          ************************************************************

Ct3       ILKNNQTRKARLSDTEIILNMVQFISAGSETTASLLAGWTYFIVANPHVYKRVVAEVREA
Ct4       ILKNNQTRKARLSDTEIILNMVQFISAGSETTASLLAGWTYFIVANPHVYKRVVAEVREA
Ct61      ILKNNQTRKARLSDTEIILNMVQFISAGSETTASLLAGWTYFIVANPHVYKRVVAESLQC
          ********************************************************  :.

Ct3       FNAKEEITWTSVGKLKYLEATLHEALRLTSPAPCNQHRVVPPAGQGKVIDGHYVPPGTTV
Ct4       FNAKEEITWTSVGKLKYLEATLHEALRLTSPAPCNQHRVVPPAGQGKVIDGHYVPPGTTV
Ct61      -------TPGRVGKLKYLEATLHEALRLTSPAPCNQHRVVPPAGQGKVIDGHYVPPGTTV
                 *   *************************************************

Ct3       AVAPWVAERHPDNFTDPETFEPERWLGAERYKNDKLHASQPFGLGVRACIGKNLSYFEAR
Ct4       AVAPWVAERHPDNFTDPETFEPERWLGAERYKNDKLHASQPFGLGVRACIGKNLSYFEAR
Ct61      AVAPWVAERHPDNFTDPETFEPERWLGAERYKNDKLHASQPFGLGVRACIGKNLSYFEAR
          ************************************************************

Ct3       LMMGHLLWHFDFAFEETKSAQAALRKWETADMLVWHVWDKPPLMVQFSEVHR
Ct4       LMMGHLLWHFDFAFEETKSAQAALRKWETADMLVWHVWDKPPLMVQFSEVHR
Ct61      LMMGHLLWHFDFAFEETKSAQAALRKWETADMLVWHVWDKPPLMVQFSEVHR
          ****************************************************

ABA3
Ct3       MQKTPERAPRERDTWYFPPDIAHDLDGIPLPESVKGEILATAWEYTRTVIPNYTNWKRYV
Ct4       MQKTPERAPRERDTWYFPPDIAHDLDGIPLPESVKGEILATAWEYTRTVIPNYTNWKRYV
Ct61      MQKTPERAPRERDTWYFPPDIAHDLDGIPLPESVKGEILATAWEYTRTVIPNYTNWKRYV
          ************************************************************

Ct3       AFMRIVVMGIIAEFKGDMLDVTLGDSVLNYSLDGVLSDLFKGTPGHTAMAREYKTFLLVS
Ct4       AFMRIVVMGIIAEFKGDMLDVTLGDSVLNYSLDGVLSDLFKGTPGHTAMAREYKTFLLVS
Ct61      AFMRIVVMGIIAEFKGDMLDVTLGDSVLNYSLDGVLSDLFKGTPGHTAMAREYKTFLLVS
          ************************************************************

Ct3       GDKSSSRRTGEFFRRYVNVLAHSPTRFFRLRDTDALCRFTIATALACNDLDDVWFTEEQF
Ct4       GDKSSSRRTGEFFRRYVNVLAHSPTRFFRLRDTDALCRFTIATALACNDLDDVWFTEEQF
Ct61      GDKSSSRRTGEFFRRYVNVLAHSPTRFFRLRDTDALCRFTIATALACNDLDDVWFTEEQF
          ************************************************************

Ct3       DLLAEMGDTMYDAVSFFKHRSEGETNSTFAYMPADLRLAAYRQCRELLWALDAAWCTRPE
Ct4       DLLAEMGDTMYDAVSFFKHRSEGETNSTFAYMPADLRLAAYRQCRELLWALDAAWCTRPE
Ct61      DLLAEMGDTMYDAVSFFKHRSEGETNSTFAYMPADLRLAAYRQCRELLWALDAAWCTRPE
          ************************************************************

Ct3       TACVTSFLRYFGGPLHLMMRRYRFVEEGLTIGREEDAEVVNQTRNHYKLWNRIDANLRHA
Ct4       TACVTSFLRYFGGPLHLMMRRYRFVEEGLTIGREEDAEVVNQTRNHYKLWNRIDANLRHA
Ct61      TACVTSFLRYFGGPLHLMMRRYRFVEEGLTIGREEDAEVVNQTRNHYKLWNRIDANLRHA
          ************************************************************

Ct3       NEREKASLERYDAIVADSEKLLIPGLAGFLEAGGDGHCDSCRYQVSYGADGEYRFGGVEL
Ct4       NEREKASLERYDAIVADSEKLLIPGLAGFLEAGGDGHCDSCRYQVSYGADGEYRFGGVEL
Ct61      NEREKASLERYDAIVADSEKLLIPGLAGFLEAGGDGHCDSCRYQVSYGADGEYRFGGVEL
          ************************************************************

Ct3       CEDCRSKWRAFLLSFPERAAKVFPELAGTYKIAITSSNKLESSGDGPLVLVPAA
Ct4       CEDCRSKWRAFLLSFPERAAKVFPELAGTYKIAITSSNKLESSGDGPLVLVPAA
Ct61      CEDCRSKWRAFLLSFPERAAKVFPELAGTYKIAITSSNKLESSGDGPLVLVPAA
          ******************************************************

ABA4
Ct3       MSTPHQFSDKVIAITGAASGIGLATAHLLASRGAKLSLTDTNKEGLIEVQLNIQTQYPQV
Ct4       MSTPHQFSDKVIAITGAASGIGLATAHLLASRGAKLSLTDTNKEGLIEVQLNIQTQYPQV
Ct61      MSTPHQFSDKVIAITGAASGIGLATAHLLASRGAKLSLTDTNKEGLIEVQLNIQTQYPQV
          ************************************************************
```

```
Ct3     EVLISAADVTDYEQIEHWTVNTVDHFGKLDGAANLAGVIPKSVGKKGLIDQDFEEWDFVM
Ct4     EVLISAADVTDYEQIEHWTVNTVDHFGKLDGAANLAGVIPKSVGKKGLIDQDFEEWDFVM
Ct61    EVLISAADVTDYEQIEHWTVNTVDHFGKLDGAANLAGVIPKSVGKKGLIDQDFEEWDFVM
        ************************************************************

Ct3     GVNLKGVMHCLKAQLSVIQDHGSIVNASSIAGLQGRPYNGAYTAKHRRKLGYKPTRLWDA
Ct4     GVNLKGVMHCLKAQLSVIQDHGSIVNASSIAGLQGRPYNGAYTASKHAIVGLTRTAAKEV
Ct61    GVNLKGVMHCLKAQLSVIQDHGSIVNASSIAGLQGRPYNGAYTASKHAIVGLTRTAAKEV
        ********************************************.::  :* . *   :.

Ct3     --------QASPRK----------------------------------------------
Ct4     GLRGIRVNAVCPGRIDTPMSRASAEAGIQADAALGRSGKPEEVASLIAFLLSNESTYITG
Ct61    GLRGIRVNAVCP------------------------------------------------
                 ..*

Ct3     -----------
Ct4     NAVSIDGGWNC
Ct61    -----------
```

BOT1

```
Ct3     MGLLSDVVNVVHDYLPQTPLAWAAFALGAFALYSIQLVVRRLYFHPLAKIPGPFLARTTY
Ct4     MGLLSDVVNVVHDYLPQTPLAWAAFALGAFALYSIQLVVRRLYFHPLAKIPGPFLARTTY

Ct61    MGLLSDVVNVVHDYLPQTPLAWAAFALGAFALYSIQLVVRRLYFHPLAKIPGPFLARTTY
        ************************************************************

Ct3     WYEFYQDIILGGMYVKNYAALHEKYGPVIRASPDRVHVSDPDFFHEVYSSGSKYMKDPAF
Ct4     WYEFYQDIILGGMYVKNYAALHEKYGPVIRASPDRVHVSDPDFFHEVYSSGSKYMKDPAF
Ct61    WYEFYQDIILGGMYVKNYAALHEKYGPVIRASPDRVHVSDPDFFHEVYSSGSKYMKDPAF
        ************************************************************

Ct3     FQSSGGIPEALPAIVDVEYHKRRRKLINDLFSAKSMEALSHLVLKVVQNALRKANEHHDE
Ct4     FQSSGGIPEALPAIVDVEYHKRRRKLINDLFSAKSMEALSHLVLKVVQNALRKANEHHDE
Ct61    FQSSGGIPEALPAIVDVEYHKRRRKLINDLFSAKSMEALSHLVLKVVQNALRKANEHHDE
        ************************************************************

Ct3     NKVLDIQRLYTGITIDTIMQVLCDRTLNFIDAKEEEPPFLATLRTFSENFFLLKHFPVLI
Ct4     NKVLDIQRLYTGITIDTIMQVLCDRTLNFIDAKEEEPPFLATLRTFSENFFLLKHFPVLI
Ct61    NKVLDIQRLYTGITIDTIMQVLCDRTLNFIDAKEEEPPFLATLRTFSENFFLLKHFPVLI
        ************************************************************

Ct3     WMALNIPKSIAAKLIPGEFEFRANINQWIRDRALEHELGVEKAEDGRKTVIDLLLRPEDG
Ct4     WMALNIPKSIAAKLIPGEFEFRANINQWIRDRALEHELGVEKAEDGRKTVIDLLLRPEDG
Ct61    WMALNIPKSIAAKLIPGEFEFRANINQWIRDRALEHELGVEKAEDGRKTVIDLLLRPEDG
        ************************************************************

Ct3     GRPLTHQAVEDETYSFAFAGTHTTSHTMSMGTYYLLSNPSKLQKLRDELKTVPKNDQGLY
Ct4     GRPLTHQAVEDETYSFAFAGTHTTSHTMSMGTYYLLSNPSKLQKLRDELKTVPKNDQGLY
Ct61    GRPLTHQAVEDETYSFAFAGTHTTSHTMSMGTYYLLSNPSKLQKLRDELKTVPKNDQGLY
        ************************************************************

Ct3     EYKTVRALPYLNACIKESLRMSSPVPGILPRLVPVGGMTWQGHYLPAGTSVSSSIHCVHN
Ct4     EYKTVRALPYLNACIKESLRMSSPVPGILPRLVPVGGMTWQGHYLPAGTSVSSSIHCVHN
Ct61    EYKTVRALPYLNACIKESLRMSSPVPGILPRLVPVGGMTWQGHYLPAGTSVSSSIHCVHN
        ************************************************************

Ct3     DPSIFPNPEQFIPERWLANENLEHYLVVFGKGSRACIGLNVAWMETYLTFSNFFTSLDMS
Ct4     DPSIFPNPEQFIPERWLANENLEHYLVVFGKGSRACIGLNVAWMETYLTFSNFFTSLDMS
Ct61    DPSIFPNPEQFIPERWLANENLEHYLVVFGKGSRACIGLNVAWMETYLTFSNFFTSLDMS
        ************************************************************

Ct3     LFETNEQTTDWTDCGNSMIKKHVMVKVNSVAS
Ct4     LFETNEQTTDWTDCGNSMIKKHVMVKVNSVAS
Ct61    LFETNEQTTDWTDCGNSMIKKHVMVKVNSVAS
        ********************************
```

BOT2

```
Ct3      MATLAPALELHNADTASSDVSSNSTYSGYDTSYTTPGEKLDTQGLELQHDAKRPPFVRIP
Ct4      MATLAPALELHNADTASSDVSSNSTYSGYDTSYTTPGEKLDTQGLELQHDAKRPPFVRIP
Ct61     MATLAPALELHNADTASSDVSSNSTYSGYDTSYTTPGEKLDTQGLELQHDAKRPPFVRIP
         ************************************************************

Ct3      DLFGSIMATKPVVNPNYFAAKARGDRWIARVMNFDKVTAARNAKVDLCFLASIWAPDASE
Ct4      DLFGSIMATKPVVNPNYFAAKARGDRWIARVMNFDKVTAARNAKVDLCFLASIWAPDASE
Ct61     DLFGSIMATKPVVNPNYFAAKARGDRWIARVMNFDKVTAARNAKVDLCFLASIWAPDASE
         ************************************************************

Ct3      DRLVMMLDWNHWVFLFDDQFDEGHLKEDPAAAAEEVRQTVAIMGGDAPVYTAESNPIRYV
Ct4      DRLVMMLDWNHWVFLFDDQFDEGHLKEDPAAAAEEVRQTVAIMGGDAPVYTAESNPIRYV
Ct61     DRLVMMLDWNHWVFLFDDQFDEGHLKEDPAAAAEEVRQTVAIMGGDAPVYTAKSNPIRYV
         ****************************************************:*******

Ct3      FQECWERLRSVSSQEMQQRWIDQHKRYFAQLLVQVDQQVSGQNFVRDVDAYMDLRRGTIG
Ct4      FQECWERLRSVSSQEMQQRWIDQHKRYFAQLLVQVDQQVSGQNFVRDVDAYMDLRRGTIG
Ct61     FQECWERLRSVSSQEMQQRWIDQHKRYFAQLLVQVDQQVSGQNFVRDVDAYMDLRRGTIG
         ************************************************************

Ct3      VYPAINLAEYGADVRLPQYVYDHPSLQECMKISADLVTLVNDVLSYRKDLELGVDHNLIS
Ct4      VYPAINLAEYGADVRLPQYVYDHPSLQECMKISADLVTLVNDVLSYRKDLELGVDHNLIS
Ct61     VYPAINLAEYGADVRLPQYVYDHPSLQECMKISADLVTLVNDVLSYRKDLELGVDHNLIS
         ************************************************************

Ct3      LLMERDSLSIQQAVDKIGDMVNDCYRRWYLALAELPSYGEKIDYKVMKFIEICRAVAQGN
Ct4      LLMERDSLSIQQAVDKIGDMVNDCYRRWYLALAELPSYGEKIDYKVMKFIEICRAVAQGN
Ct61     LLMERDSLSIQQAVDKIGDMVNDCYRRWYLALAELPSYGEKIDYKVMKFIEICRAVAQGN
         ************************************************************

Ct3      LYWSFQTGRYLGPEGHDVHETGIMHLPPIKALF
Ct4      LYWSFQTGRYLGPEGHDVHETGIMHLPPIKALF
Ct61     LYWSFQTGRYLGPEGHDVHETGIMHLPPIKALF
         *********************************

BOT3
Ct3      MEKSSPALALASEKAWEFASKADAVLRTGVSRAAPHVLAAKNKAWEGAHSIGTKLAQGNV
Ct4      MEKSSPALALASEKAWEFASKADAVLRTGVSRAAPHVLAAKNKAWEGAHSIGTKLAQGNV
Ct61     MEKSSPALALASEKAWEFASKADAVLRTGVSRAAPHVLAAKNKAWEGAHSIGTKLAQGNV
         ************************************************************

Ct3      GDIDRTEVTTILSVVLGLYFAYVFALVFYRLYLHPLAKFPGYKICAACEWYEFYCYIVKG
Ct4      GDIDRTEVTTILSVVLGLYFAYVFALVFYRLYLHPLAKFPGYKICAACEWYEFYCYIVKG
Ct61     GDIDRTEVTTILSVVLGLYFAYVFALVFYRLYLHPLAKFPGYKICAACEWYEFYCYIVKG
         ************************************************************

Ct3      GQWGNEIRKMHEKYGPIVRTSPWELSIRDPVFYDQLYVTAATRKTDMWPRGREGNGFDNS
Ct4      GQWGNEIRKMHEKYGPIVRTSPWELSIRDPVFYDQLYVTAATRKTDMWPRGREGNGFDNS
Ct61     GQWGNEIRKMHEKYGPIVRTSPWELSIRDPVFYDQLYVTAATRKTDMWPRGREGNGFDNS
         ************************************************************

Ct3      HHLSVSHDLHRIRRKHLEPFFSRQGITRIEPRIAELVMKMDKRIVGLKGSGAVVPVDHLL
Ct4      HHLSVSHDLHRIRRKHLEPFFSRQGITRIEPRIAELVMKMDKRIVGLKGSGAVVPVDHLL
Ct61     HHLSVSHDLHRIRRKHLEPFFSRQGITRIEPRIAELVMKMDKRIVGLKGSGAVVPVDHLL
         ************************************************************

Ct3      CALTGDVIGQVSSGMQAGLLDDPDFTPAWKDLMIKSTTIAPLFRCFPWINKFLQSLPTSI
Ct4      CALTGDVIGQVSSGMQAGLLDDPDFTPAWKDLMIKSTTIAPLFRCFPWINKFLQSLPTSI
Ct61     CALTGDVIGQVSSGMQAGLLDDPDFTPAWKDLMIKSTTIAPLFRCFPWINKFLQSLPTSI
         ************************************************************

Ct3      MNSVYPKGISNMMLGKTGQRNIEKIKSDIATSKKDLSGVSVFHHLLSSDVPESEKSTDRL
Ct4      MNSVYPKGISNMMLGKTGQRNIEKIKSDIATSKKDLSGVSVFHHLLSSDVPESEKSTDRL
Ct61     MNSVYPKGISNMMLGKTGQRNIEKIKSDIATSKKDLSGVSVFHHLLSSDVPESEKSTDRL
         ************************************************************

Ct3      RAESMILLLLAGTLAGAHTLTFVVFYVLQNPQIEKRLRTELQPVFKGYPNKMPTWAELERL
Ct4      RAESMILLLLAGTLAGAHTLTFVVFYVLQNPQIEKRLRTELQPVFKGYPNKMPTWAELERL
Ct61     RAESMILLLLAGTLAGAHTLTFVVFYVLQNPQIEKRLRTELQPVFKGYPNKMPTWAELERL
```

****

Ct3 PYLRGCIKEALRLNGLVGNLARCSPDEDIQFRQWVIPKKTPVGMSIYAMHFDPSVFPEPE
Ct4 PYLRGCIKEALRLNGLVGNLARCSPDEDIQFRQWVIPKKTPVGMSIYAMHFDPSVFPEPE
Ct61 PYLRGCIKEALRLNGLVGNLARCSPDEDIQFRQWVIPKKTPVGMSIYAMHFDPSVFPEPE
****

Ct3 AFKPERWVGQYDRNMDRNFVPFTKGSRSCLGINLAWAELYLASAMLFRPGGPKLVLHNAD
Ct4 AFKPERWVGQYDRNMDRNFVPFTKGSRSCLGINLAWAELYLASAMLFRPGGPKLVLHNAD
Ct61 AFKPERWVGQYDRNMDRNFVPFTKGSRSCLGINLAWAELYLASAMLFRPGGPKLVLHNAD
****

Ct3 ESDIKISRDYIMGFARADAKDIRIKVE
Ct4 ESDIKISRDYIMGFARADAKDIRIKVE
Ct61 ESDIKISRDYIMGFARADAKDIRIKVE
****

BOT4

Ct3 MDMAEYNVTTSTPEPKAGWPAVERLLGAYTTPETWSWQVVAGCLATIILCRFVAVWSYNL
Ct4 MDMAEYNVTTSTPEPKAGWPAVERLLGAYTTPETWSWQVVAGCLATIILCRFVAVWSYNL
Ct61 MDMAEYNVTTSTPEPKAGWPAVERLLGAYTTPETWSWQVVAGCLATIILCRFVAVWSYNL
****

Ct3 WFHPLARFPGPLLGRCSLIFRFMHSSRGRIHSAIADAHKKYGDIVRIAPNELSFASVESW
Ct4 WFHPLARFPGPLLGRCSLIFRFMHSSRGRIHSAIADAHKKYGDIVRIAPNELSFASVESW
Ct61 WFHPLARFPGPLLGRCSLIFRFMHSSRGRIHSAIADAHKKYGDIVRIAPNELSFASVESW
****

Ct3 KAIYGHPTGGRPIAPKGPFYEVFAAGFSSKCVGSERDPKKHSAMRKMLNPAFSQRGLLEQ
Ct4 KAIYGHPTGGRPIAPKGPFYEVFAAGFSSKCVGSERDPKKHSAMRKMLNPAFSQRGLLEQ
Ct61 KAIYGHPTGGRPIAPKGPFYEVFAAGFSSKCVGSERDPKKHSAMRKMLNPAFSQRGLLEQ
****

Ct3 EEIISGIIDKFVSILGEKAGPGTKGLNMTKWYEMNSFDILGEMAFGESFHSLDTGVPHFW
Ct4 EEIISGIIDKFVSILGEKAGPGTKGLNMTKWYEMNSFDILGEMAFGESFHSLDTGVPHFW
Ct61 EEIISGIIDKFVSILGEKAGPGTKGLNMTKWYEMNSFDILGEMAFGESFHSLDTGVPHFW
****

Ct3 ADIVLEHLYVITLFDNLRRIGWLAKLAGLLVPASIVTSNQNSNYSRQQVEKRLKTQESRN
Ct4 ADIVLEHLYVITLFDNLRRIGWLAKLAGLLVPASIVTSNQNSNYSRQQVEKRLKTQESRN
Ct61 ADIVLEHLYVITLFDNLRRIGWLAKLAGLLVPASIVTSNQNSNYSRQQVEKRLKTQESRN
****

Ct3 DFVSLLVDKVRAGEVSKEEMTAHVSTLTIAGGETVATTLSSITFFLAQNPEKFERLTKEI
Ct4 DFVSLLVDKVRAGEVSKEEMTAHVSTLTIAGGETVATTLSSITFFLAQNPEKFERLTKEI
Ct61 DFVSLLVDKVRAGEVSKEEMTAHVSTLTIAGGETVATTLSSITFFLAQNPEKFERLTKEI
****

Ct3 RAAFKSYKEINAVKAQQLPYLQAVINEGLRLFPPASNGASRVSPGFSLHGKYIPEGTEIN
Ct4 RAAFKSYKEINAVKAQQLPYLQAVINEGLRLFPPASNGASRVSPGFSLHGKYIPEGTEIN
Ct61 RAAFKSYKEINAVKAQQLPYLQAVINEGLRLFPPASNGASRVSPGFSLHGKYIPEGTEIN
****

Ct3 VSPWSITHNPKYFSDPWDFKPERWLDPNSTDNRDANRPFLLGPRDCLGRNFALMELNLVL
Ct4 VSPWSITHNPKYFSDPWDFKPERWLDPNSTDNRDANRPFLLGPRDCLGRNFALMELNLVL
Ct61 VSPWSITHNPKYFSDPWDFKPERWLDPNSTDNRDANRPFLLGPRDCLGRNFALMELNLVL
****

Ct3 AKLLWSYDMELVNKEVNFLEQSTVHVLWWKPALFVRWHRPQSTSS
Ct4 AKLLWSYDMELVNKEVNFLEQSTVHVLWWKPALFVRWHRPQSTSS
Ct61 AKLLWSYDMELVNKEVNFLEQSTVHVLWWKPALFVRWHRPQSTSS
****

BOT5

Ct3 MATIPFFFSLVLFLWHYFAYKLQKKQGGQGYIATEDAASKPDSPQGDDEVLPVHFIDQAA
Ct4 MATIPFFFSLVLFLWHYFAYKLQKKQGGQGYIATEDAASKPDSPQGDDEVLPVHFIDQAA
Ct61 MATIPFFFSLVLFLWHYFAYKLQKKQGGQGYIATEDAASKPDSPQGDDEVLPVHFIDQAA
****

Ct3 IVRTSIINYTFRYNDVLDASKLHRGLLHLLQIPGWNKLGGRLRATSGGKLEIHVPKTFSR

```
Ct4      IVRTSIINYTFRYNDVLDASKLHRGLLHLLQIPGWNKLGGRLRATSGGKLEIHVPKTFSR
Ct61     IVRTSIINYTFRYNDVLDASKLHRGLLHLLQIPGWNKLGGRLRATSGGKLEIHVPKTFSR
         ************************************************************

Ct3      SRPAVKFSHVDLSDTRIEIHHLASQLPVSTGSLPSVQEGCHAFRTFALPADLPNNIDHYW
Ct4      SRPAVKFSHVDLSDTRIEIHHLASQLPVSTGSLPSVQEGCHAFRTFALPADLPNNIDHYW
Ct61     SRPAVKFSHVDLSDTRIEIHHLASQLPVSTGSLPSVQEGCHAFRTFALPADLPNNIDHYW
         ************************************************************

Ct3      KNDQPLLCLHVTSFANATLVGFTFPHSLSDAMGTSELLKAWSNVVAGKSCLVKPLHGTHV
Ct4      KNDQPLLCLHVTSFANATLVGFTFPHSLSDAMGTSELLKAWSNVVAGKSCLVKPLHGTHV
Ct61     KNDQPLLCLHVTSFANATLVGFTFPHSLSDAMGTSELLKAWSNVVAGKSCLVKPLHGTHV
         ************************************************************

Ct3      DVLGDVGTAFDKKASGGEFVLERQQTRAFSLLSFIVRYVWDVTTRRSIRTRHIYLPAKYM
Ct4      DVLGDVGTAFDKKASGGEFVLERQQTRAFSLLSFIVRYVWDVTTRRSIRTRHIYLPAKYM
Ct61     DVLGDVGTAFDKKASGGEFVLERQQTRAFSLLSFIVRYVWDVTTRRSIRTRHIYLPAKYM
         ************************************************************

Ct3      RHLRHSVEEELRRKNRGVAPFVSDGDLITAWGSRMVISSSPWRNCSAVICNVFDLRGRLK
Ct4      RHLRHSVEEELRRKNRGVAPFVSDGDLITAWGSRMVISSSPWRNCSAVICNVFDLRGRLK
Ct61     RHLRHSVEEELRRKNRGVAPFVSDGDLITAWGSRMVISSSPWRNCSAVICNVFDLRGRLK
         ************************************************************

Ct3      NTFTPGGTYLQNLILPATTVLSRDETANATTAQIAWGLRKAILEQTEDVQARRLMRLARQ
Ct4      NTFTPGGTYLQNLILPATTVLSRDETANATTAQIAWGLRKAILEQTEDVQARRLMRLARQ
Ct61     NTFTPGGTYLQNLILPATTVLSRDETANATTAQIAWGLRKAILEQTEDVQARRLMRLARQ
         ************************************************************

Ct3      WFASMGMMPLFAGWDSRVIACTNWTKAKFLDAADFGPTALVSLGNHKPNGPTRKAVAEGA
Ct4      WFASMGMMPLFAGWDSRVIACTNWTKAKFLDAADFGPTALVSLGNHKPNGPTRKAVAEGA
Ct61     WFASMGMMPLFAGWDSRVIACTNWTKAKFLDAADFGPTALVSLGNHKPNGPTRKAVAEGA
         ************************************************************

Ct3      RPGRPVMYWGTTLSVTDSPRDTFVIYGKDAAGNYWIHAYLREETWDLIQKELLSFR
Ct4      RPGRPVMYWGTTLSVTDSPRDTFVIYGKDAAGNYWIHAYLREETWDLIQKELLSFR
Ct61     RPGRPVMYWGTTLSVTDSPRDTFVIYGKDAAGNYWIHAYLREETWDLIQKELLSFR
         ********************************************************

BOT6 (Zn clus)
Ct3      MYGTLSFSKGNKKSSAEFHAFSEVHKRRLRQPACDECRIARVKCRGKSDGEKCARCQGTG
Ct4      MYGTLSFSKGNKKSSAEFHAFSEVHKRRLRQPACDECRIARVKCRGKSDGEKCARCQGTG
Ct61     MYGTLSFSKGNKKSSAEFHAFSEVHKRRLRQPACDECRIARVKCRGKSDGEKCARCQGTG
         ************************************************************

Ct3      RACTYNSTRRRSQASGKPAEPTEPTHTTDDTDEDTGDKDGDGTPSAPSALEATASPQGPS
Ct4      RACTYNSTRRRSQASGKPAEPTEPTHTTDDTDEDTGDKDGDGTPSAPSALEATASPQGPS
Ct61     RACTYNSTRRRSQASGKPAEPTEPTHTTDDTDEDTGDKDGDGTPSAPSALEATASPQGPS
         ************************************************************

Ct3      SLPHDAHTKWWECRFEPFDLFVDPGADLAVDEAQHAAPRKHVDGDSAAGSVNDGDGVLHL
Ct4      SLPHDAHTKWWECRFEPFDLFVDPGADLAVDEAQHAAPRKHVDGDSAAGSVNDGDGVLHL
Ct61     SLPHDAHTKWWECRFEPFDLFVDPGADLAVDEAQHAAPRKHVDGDSAAGSVNDGDGVLHL
         ************************************************************

Ct3      ADLELASAVIGHHDAHHYGFPIPCSDFQAKIAPSTSSRSSPPTPSLVWSTMSGHGEHQRP
Ct4      ADLELASAVIGHHDAHHYGFPIPCSDFQAKIAPSTSSRSSPPTPSLVWSTMSGHGEHQRP
Ct61     ADLELASAVIGHHDAHHYGFPIPCSDFQAKIAPSTSSRSSPPTPSLVWSTMSGHGEHQRP
         ************************************************************

Ct3      PSLSSDMQLDHAASLPHNPDAQDDATHASTLAEFSDLIDVMQFAEGPSISGPATPASSSC
Ct4      PSLSSDMQLDHAASLPHNPDAQDDATHASTLAEFSDLIDVMQFAEGPSISGPATPASSSC
Ct61     PSLSSDMQLDHAASLPHNPDAQDDATHASTLAEFSDLIDVMQFAEGPSISGPATPASSSC
         ************************************************************

Ct3      TCLQDLTATLFSLRSRPDKTQVDHFLLLFKQVMQKWEAVETCARACRVSRSFALLVLMNV
Ct4      TCLQDLTATLFSLRSRPDKTQVDHFLLLFKQVMQKWEAVETCARACRVSRSFALLVLMNV
Ct61     TCLQDLTATLFSLRSRPDKTQVDHFLLLFKQVMQKWEAVETCARACRVSRSFALLVLMNV
         ************************************************************
```

```
Ct3        QELVTLLLEATSSANTSSGTAGRRESVLAISMGTFTVEDGADQRIIARMLLAVRMKELHS
Ct4        QELVTLLLEATSSANTSSGTAGRRESVLAISMGTFTVEDGADQRIIARMLLAVRMKELHS
Ct61       QELVTLLLEATSSANTSSGTAGRRESVLAISMGTFTVEDGADQRIIARMLLAVRMKELHS
           ************************************************************

Ct3        FITRISSQMKLAGLDDIFTDFHHQIEMLRRAFTL
Ct4        FITRISSQMKLAGLDDIFTDFHHQIEMLRRAFTL
Ct61       FITRISSQMKLAGLDDIFTDFHHQIEMLRRAFTL
           **********************************

BOT7 (adh short)
Ct3        MPDAKPIYNLPEDAVWFITGCSSGMGLALAQIVASKPTQRVVATARSVSKLAGKLPDSPR
Ct4        MPDAKPIYNLPEDAVWFITGCSSGMGLALAQIVASKPTQRVVATARSVSKLAGKLPDSPR
Ct61       MPDAKPIYNLPEDAVWFITGCSSGMGLALAQIVASKPTQRVVATARSVSKLAGKLPDSPR
           ************************************************************

Ct3        VHLTSLDVTSADSITAAFDSAVAKFGRIDVVVNNAGYGLMGDTESFVINAEDHDKARKVV
Ct4        VHLTSLDVTSADSITAAFDSAVAKFGRIDVVVNNAGYGLMGDTESFVINAEDHDKARKVV
Ct61       VHLTSLDVTSADSITAAFDSAVAKFGRIDVVVNNAGYGLMGDTESFVINAEDHDKARKVV
           ************************************************************

Ct3        ETNFWGTAQVSAHAVRVFRDENPKSGQIGGVVLNVTSIGGFAGFPGSAFYHASKFAVEGY
Ct4        ETNFWGTAQVSAHAVRVFRDENPKSGQIGGVVLNVTSIGGFAGFPGSAFYHASKFAVEGY
Ct61       ETNFWGTAQVSAHAVRVFRDENPKSGQIGGVVLNVTSIGGFAGFPGSAFYHASKFAVEGY
           ************************************************************

Ct3        TESLSKEVRSDWNIHFSIIEPGGTKTNFAGDSMAWFSAHPAYAAPDTPTRLLEGYVKSPD
Ct4        TESLSKEVRSDWNIHFSIIEPGGTKTNFAGDSMAWFSAHPAYAAPDTPTRLLEGYVKSPD
Ct61       TESLSKEVRSDWNIHFSIIEPGGTKTNFAGDSMAWFSAHPAYAAPDTPTRLLEGYVKSPD
           ************************************************************

Ct3        MQATWAPSENV-AAAMYEVVARKQAIPIRFPTGAPAWSVIKAEVEEVDKELLEIKELSFS
Ct4        MQATWAPSENV-AAAMYEVVARKQAIPIRFPTGAPAWSVIKAEVEEVDKELLEIKELSFS
Ct61       MQATWAPSENVAAAAMYEVVARKQAIPIRFPTGAPAWSVIKAEVEEVDKELLEIKELSFS
           *********** ************************************************

Ct3        VDDGSINKSGDFLKQKF
Ct4        VDDGSINKSGDFLKQKF
Ct61       VDDGSINKSGDFLKQKF
           *****************
```

Supplementary Figure 5

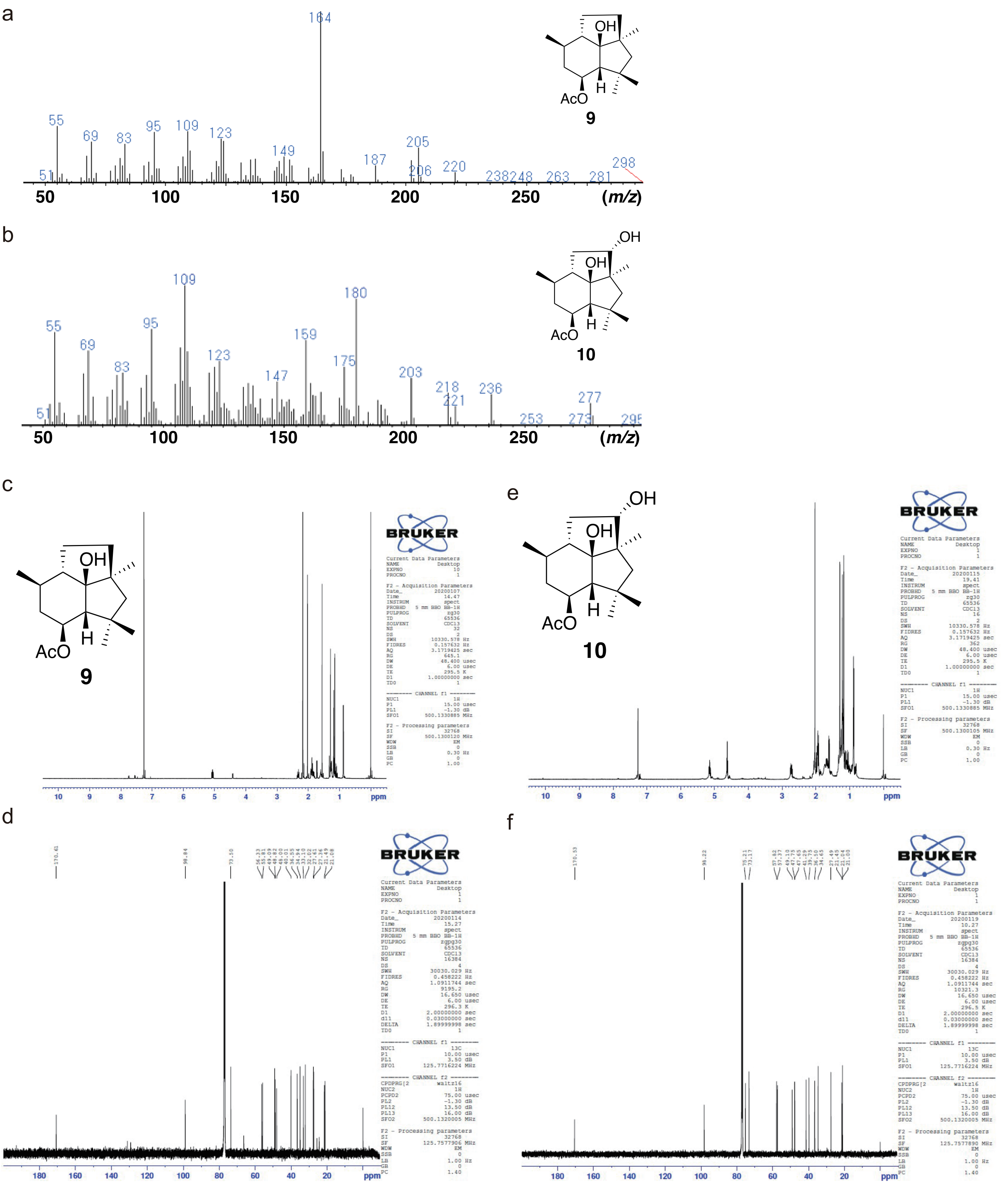

Supplementary Figure 5: Intermediate metabolites in BOT biosynthesis in Ct3. GC-MS spectra of **9** (**a**) and **10** (**b**). **c** 1H-NMR spectra of **9**. **d** 13C-NMR spectra of **9**. **e** 1H-NMR spectra of **10**. **f** 13C-NMR spectra of **10**.

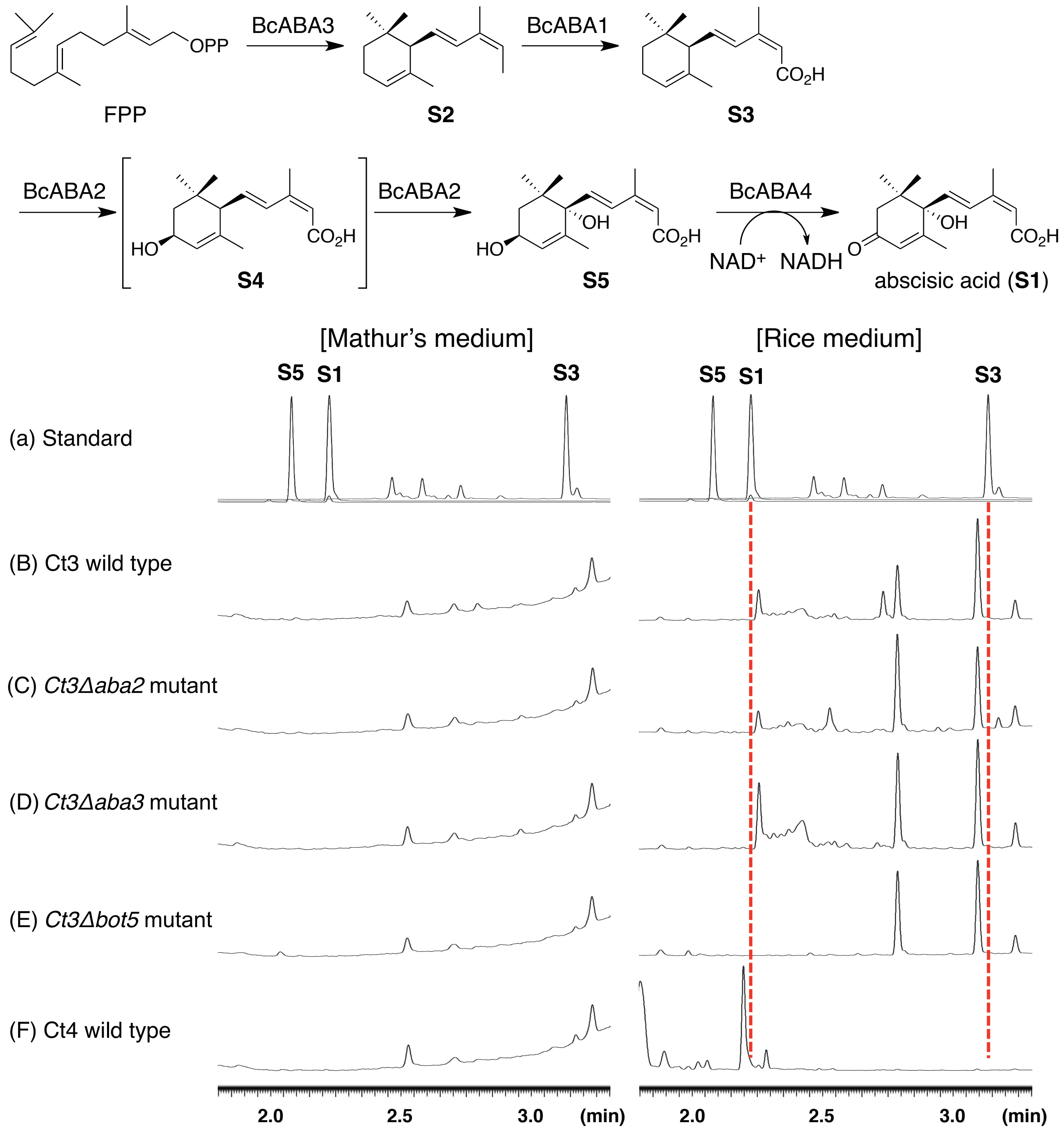

Supplementary Figure 6: Measurements of ABA and the intermediate metabolites in Mathurs media or Rice media. UPLC profiles (264 nm). (**a**) compounds, **S1**, **S3**, and **S5**, and metabolites from (**b**) Ct3 wild type strain, (**c**) Ct3*Δaba2* mutant, (**d**) Ct3*Δaba3* mutant, (**e**) Ct3*Δbot5* mutant, and (**f**) Ct4 wild type strain.

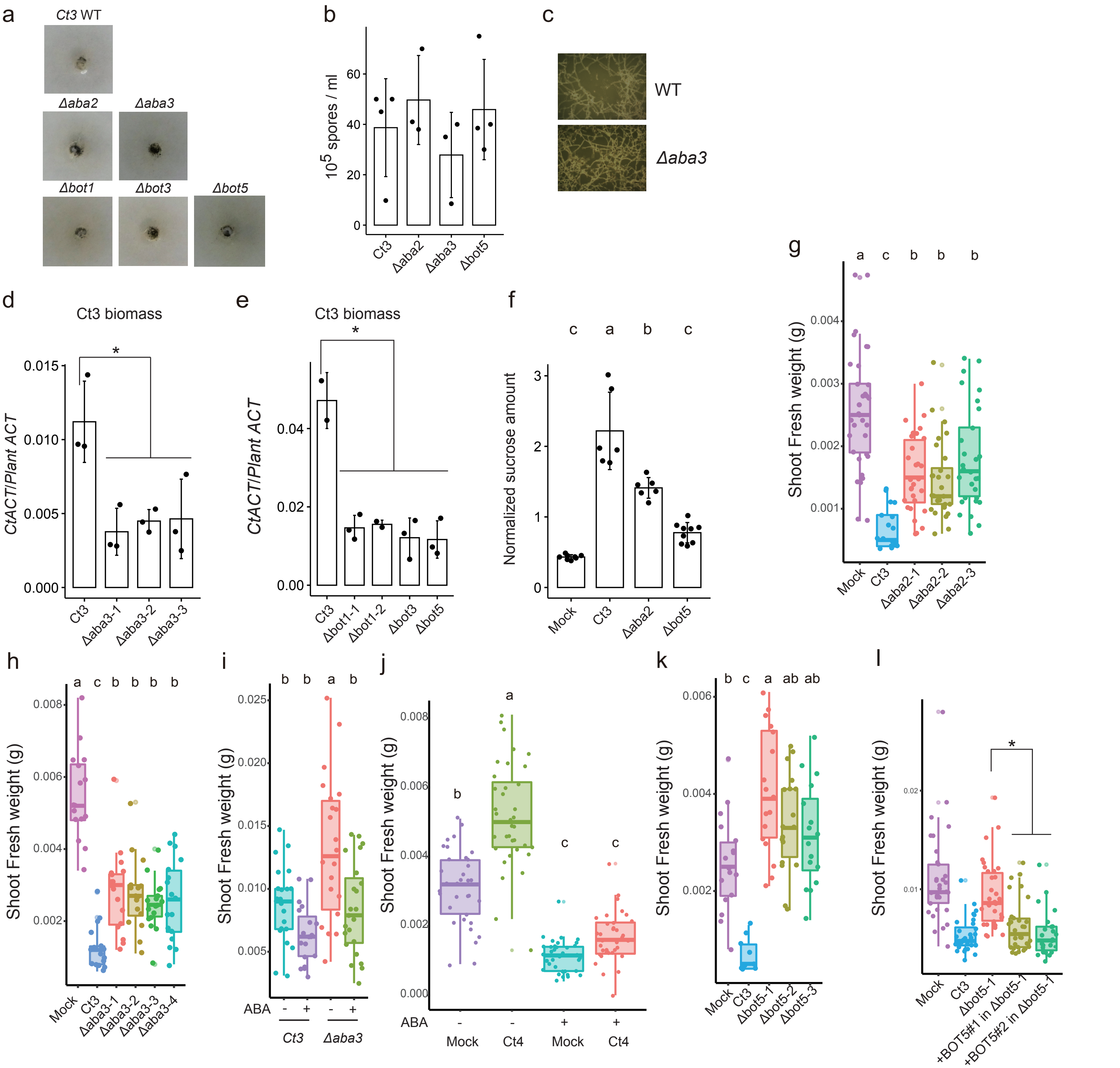

Supplementary Figure 7: Fungal ABA and BOT biosynthesis genes are required for root colonization by Ct3 and its virulence. **a** Fungal growth on low Pi media. Hyphae of Ct3WT, *Δaba2*, *Δaba3*, *Δbot1*, *Δbot3*, and *Δbot5* were transferred to low Pi media (without sucrose) and incubated for three days. **b** Measurements of fungal spores generated from fungal strains grown in Mathurs nutrient media (±SD, Ct3: n=4, *Δaba2*: n=3, *Δaba3*: n=3, *Δbot5*: n=4). **c** Spore germination and hyphal growth on glass slides. The same spore amount of Ct3WT or *Δaba3* were placed on glass slides and incubated for 2 days, after which pictures were taken. **d, e** Fungal biomass measurements in *A. thaliana* roots under low Pi (24 dpi). qRT-PCR analyses were used to measure fungal biomass as estimated by relative expression between *CtACTIN* and *Plant ACTIN* (*Ct ACTIN*/*Plant ACTIN*) (±SD, n =3, $p < 0.05$, two tailed t-test, see also source Data File for exact p value). **f** LC/MS analyses for sucrose measurements for *A. thaliana* upon fungal colonization under low Pi (Log2 (normalized)). Different letters indicate significantly different statistical groups (ANOVA, Tukey-HSD test, $p < 0.05$, ±SD, Mock: n=8, Ct3: n=6, *Δaba2*: n=6, *Δbot5*: n=9). **g, h** Measurement of *A. thaliana* SFW upon Ct3 root colonization at 24 dpi under low Pi ((**g**) Mock: n=17, Ct3: n=9, *Δaba2-1*: n=11, *Δaba2-2*: n=16, *Δaba2-3*: n=14, *Δ aba2-4:* n=14. (**h**) Mock: n=16, Ct3: n=17, *Δaba3-1*: n=17, *Δaba3-2*: n=16, *Δaba3-3*: n=18, *Δaba3-4*: n=17). Different letters indicate significantly different statistical groups (ANOVA, Tukey-HSD test, $p < 0.05$). **i** Quantitative measurement of *A. thaliana* SFW upon fungal root colonization at 24 dpi with and without ABA treatment (Ct3: n=23, Ct3_ABA: n=19, *Δaba3-1*: n=22, *Δ aba3-1*_ABA: n=22). Ct3 or Ct3*Δaba3* were incubated with *A. thaliana* for 24 dpi on low Pi media with 10 μM ABA. Different letters indicate significantly different statistical groups (ANOVA, Tukey-HSD test, $p < 0.05$). **j** Measurement of *A. thaliana* SFW upon fungal root colonization at 24 dpi with and without ABA treatment (Mock: n=34, Ct4: n=36, Mock_ABA: n=36, Ct4_ABA: n=36). Ct4 were incubated with *A. thaliana* for 24 dpi on low Pi media with 10 μM ABA. **k** Measurement of *A. thaliana* SFW upon fungal root colonization under low Pi. Different letters indicate significantly different statistical groups (ANOVA, Tukey-HSD test, $p < 0.05$). **l** Quantitative measurement of *A. thaliana* SFW upon fungal root colonization at 24 dpi under normal Pi (*Δbot5-1* vs BOT5#1 in *Δbot5-1*: p= 0.000118, *Δbot5-1* vs BOT5#2 in *Δbot5-1*: p <.0001, two tailed t-test) (Mock: n=33, Ct3: n=32, *Δbot5-1*; n=34, BOT5# in *Δbot5-1*: n=30, BOT5#2 in *Δbot5-1*; n=25).

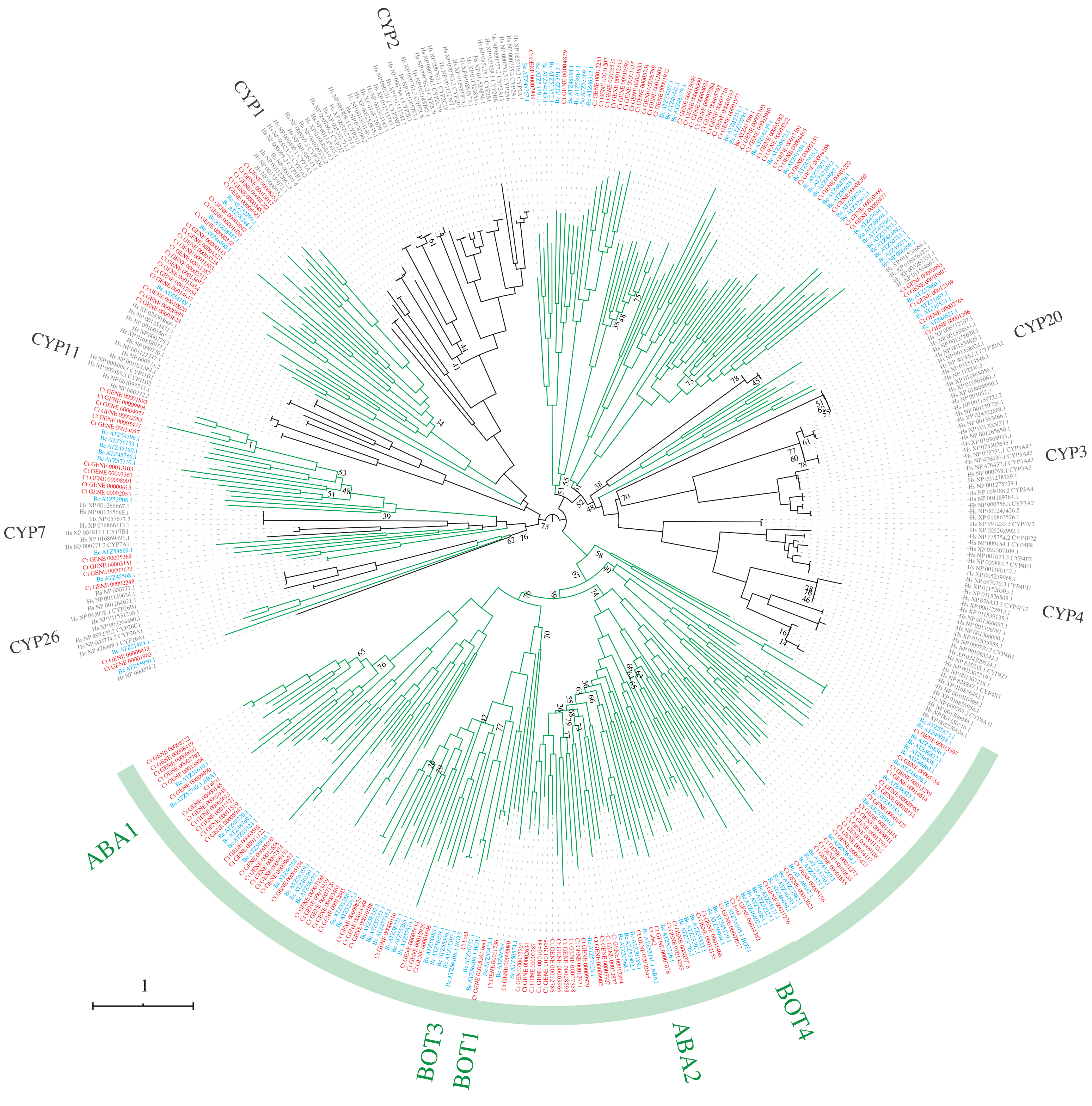

Supplementary Figure 8a Phylogenetic tree of the genes in cytochrome C P450 families. 414 genes of *C. tofieldiae* Ct3, *B. cinerea* B05.10, and *H. sapiens* are used for the ML analysis with IQ-TREE version 1.6.11. Branches with green and black indicate the lineages of fungi and humans, respectively. Red letters indicate *C. tofieldiae* genes, blue indicates *B. cinerea,* and gray indicates *H. sapiens*. ABA, BOT, and CYP genes are shown notably. Note that all the ABA and BOT genes are included in a large monophyletic clade of fungi (green arc). The number at each node corresponds to the ultrafast bootstrap values support from the ML analysis using IQ- TREE (Only less than 80% were indicated). The scale bar represents substitutions per site. Root of the tree is based on the option of root mid-point of iTOL v5.6.

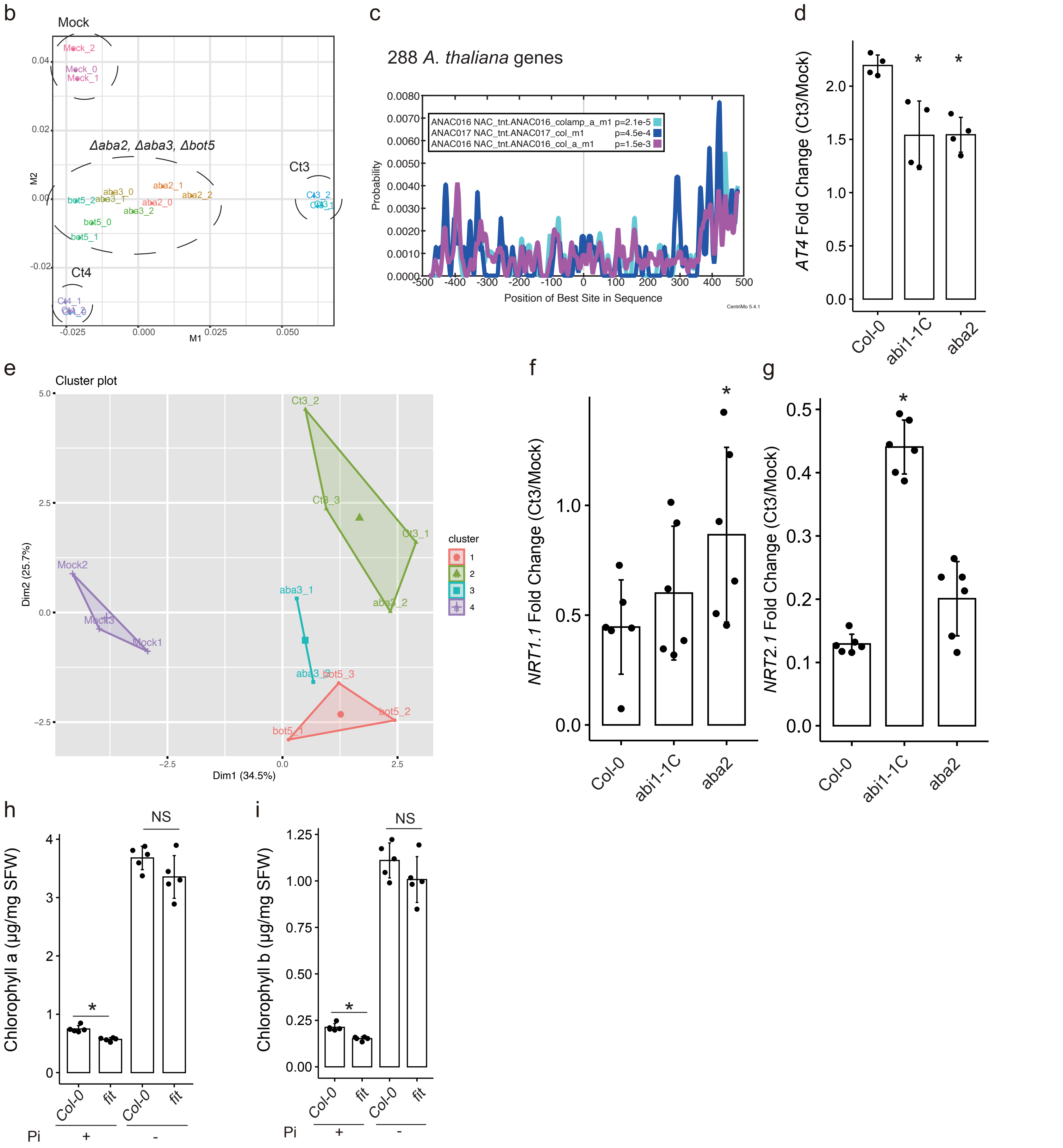

Supplementary Figure 8: Fungal ABA and BOT biosynthesis genes suppress the expression of nutrient uptake *A. thaliana* genes while activating PSR genes. **b** MDS chart generated from plant transcriptome profile during association with mock (M), Ct3, *Δaba2*, *Δaba3*, *Δbot5*, and Ct4 under low Pi. The three independent biological replicates are shown. **c** Motif analysis against 1,000 bp upstream sequences of 288 up-regulated *A. thaliana* genes during root colonization by Ct3 compared to *Δaba2*, *Δaba3*, *Δbot5*, and Ct4. **d** *AtAT4* gene fold change under low Pi. The fold change Ct3/mock is calculated by relative expression of *AtAT4*. Asterisks indicate significantly different means between Col-0 and the corresponding mutants (±SD, n = 3, $p < 0.05$, two tailed t-test). **e** PCA analysis of shoot mineral nutrient composition (ionome) in plants grown in normal Pi treated with either mock, Ct3, *Δaba2*, *Δaba3*, or *Δbot5*. The samples were separated into four different clusters. **f** *AtNRT1.1* gene fold change under low Pi. Asterisks indicate significantly different means between Col-0 and the corresponding mutants (n = 3, $p < 0.05$, two tailed t-test). **g** *AtNRT2.1* gene fold change under low Pi. Asterisks indicate significantly different means between Col-0 and the corresponding mutants (±SD, n = 3, $p < 0.05$, two tailed t-test). **h, i** Data of plant shoot chlorophyll a or b in Col-0 and *fit* under normal and low Pi. Asterisks indicate significantly different means between Col-0 and *fit* plants (±SD, $p < 0.01$, two-tailed t-test).

Supplementary Table 1: **Background information regarding *Colletotrichum tofieldiae* and KHC (Originally named as KHC23) isolates and the Ct3 mutants used in this study.**

Isolate ID	Species	Accession Number (a)	Location	Host plant	Publication
Ct61	*C. tofieldiae*	Ct0861	Spain	*A. thaliana*	3
Ct127	*C. tofieldiae*	CBS 127615	Portugal	*Agapanthus sp.*	21
Ct130	*C. tofieldiae*	CBS 130851	Germany	*Semele gayae*	21
Ct49	*C. tofieldiae*	CBS 495.85	Switzerland	*Tofieldia calyculata*	21
Ct3	*C. tofieldiae*	MAFF 712333	Japan	*Ornithogalum dubium*	24
Ct4	*C. tofieldiae*	MAFF 712334	Japan	*Ornithogalum dubium*	24
Ct3Δaba2-1	*C. tofieldiae*	MAFF 712333	Japan	*Ornithogalum dubium*	this study
Ct3Δaba2-2	*C. tofieldiae*	MAFF 712333	Japan	*Ornithogalum dubium*	this study
Ct3Δaba2-3	*C. tofieldiae*	MAFF 712333	Japan	*Ornithogalum dubium*	this study
Ct3Δaba3-1	*C. tofieldiae*	MAFF 712333	Japan	*Ornithogalum dubium*	this study
Ct3Δaba3-2	*C. tofieldiae*	MAFF 712333	Japan	*Ornithogalum dubium*	this study
Ct3Δaba3-3	*C. tofieldiae*	MAFF 712333	Japan	*Ornithogalum dubium*	this study
Ct3Δaba3-4	*C. tofieldiae*	MAFF 712333	Japan	*Ornithogalum dubium*	this study
Ct3Δbot1-1	*C. tofieldiae*	MAFF 712333	Japan	*Ornithogalum dubium*	this study
Ct3Δbot1-2	*C. tofieldiae*	MAFF 712333	Japan	*Ornithogalum dubium*	this study
Ct3Δbot3	*C. tofieldiae*	MAFF 712333	Japan	*Ornithogalum dubium*	this study

Ct3Δbot5-1	*C. tofieldiae*	MAFF 712333	Japan	*Ornithogalum dubium*	this study
Ct3Δbot5-2	*C. tofieldiae*	MAFF 712333	Japan	*Ornithogalum dubium*	this study
Ct3Δbot5-3	*C. tofieldiae*	MAFF 712333	Japan	*Ornithogalum dubium*	this study
Ct3BOT5#1 in Δ bot5-1	*C. tofieldiae*	MAFF 712333	Japan	*Ornithogalum dubium*	this study
Ct3BOT5#2 in Δ bot5-1	*C. tofieldiae*	MAFF 712333	Japan	*Ornithogalum dubium*	this study
Ci	*C. incanum*	MAFF 238704	Japan	*Raphanus sativus*	21
KHC (KHC23) b)	*C. higginsianum*	MAFF 245053	Japan	*Scopolia japonica*	this study

a) CBS: Culture Collection of the Centraalbureau voor Schimmelcultures, Fungal Biodiversity Centre, Utrecht, The Netherlands.

MAFF: MAFF Genebank Project, Ministry of Agriculture, Forestry

b) *C. higginsianum* was originally identified as C*. tofieldiae*.

Supplementary Table 2: Genome sequencing and assembly statistics of *C. tofieldiae* isolates and KHC

Assembly Statistics	Illumina +454	Pacbio	Pacbio + Illumina	Pacbio	Pacbio
Isolate	Ct61[a]	Ct61 (PacBio)	Ct4	Ct3	KHC
Genome (bp)	52836184	52991954	53983402	54252643	49069799
Number of Scaffolds	1048	28	27	19	27
Largest scaffold (bp)	861708	6294542	6028009	6503204	5997240
Smallest scaffold (bp)	201	19730	323670	8543	4021
Genome N50 (bp)	209340	4211846	3719715	6011218	4389193
GC content (%)	45.7	50.3	52.1	48.6	50.6
Number of CDS	12501	15617	14573	14916	14359
Number of tRNA	389	390	370	418	380
Number of rRNA	73	124	70	86	113
Candidate secreted proteins	1391	1397	1446	1481	1426
Candidate effector proteins	430	490	496	507	475
Carbohydrate-active enzymes	720	710	729	733	679
Candidate secondary metabolites	59	59	59	56	77
BUSCO genome mode	97.6	88.5	97.4	97.2	93.6
BUSCO protein mode	94.7	86	95	94.4	90.5

a) This genome assembly has been reported in Hacquard et al. 2016[21].

Supplementary Table 3: The information of ABA and BOT genes based on *B. cinerea* (**a**) and the bit scores of BLASTP results between ABA and BOT genes of Ct3 and *B. cinerea* (**b**)

Supplementary Table 3a

Product	Gene (*B. cinerea*)	Gene (*C. tofieldiae*)	putative function	biosynthetic role
abscisic acid	*BcABA1*	*CtABA1*	cytochrome P450	tailoring enzyme
	BcABA2	*CtABA2*	cytochrome P450	tailoring enzyme
	BcABA3	*CtABA3*	alpha-ionylideneethane synthase	sesquiterpene core formation
	BcABA4	*CtABA4*	short-chain dehydrogenase/reductase	tailoring enzyme
botrydial	*BcBOT1*	*CtBOT1*	cytochrome P450	tailoring enzyme
	BcBOT2	*CtBOT2*	presilphiperfolan-8-beta-ol synthase	sesquiterpene core formation
	BcBOT3	*CtBOT3*	cytochrome P450	tailoring enzyme
	BcBOT4	*CtBOT4*	cytochrome P450	tailoring enzyme
	BcBOT5	*CtBOT5*	acetyltranasferase	tailoring enzyme
	BcBOT6	*CtBOT6*	transcription factor	-
	BcBOT7	*CtBOT7*	deydrogenase	tailoring enzyme

Supplementary Table 3b

	Database（*Botrytis cinerea* B05.10 proteome）										
Query	BcABA1	BcABA2	BcABA3	BcABA4	BcBOT1	BcBOT2	BcBOT3	BcBOT4	BcBOT5	BcBOT6	BcBOT7
Ct3ABA1	**619**	114			108		87.0	91.3			
Ct3ABA2	105	**570**			157		122	222			
Ct3ABA3			**548**								
Ct3ABA4				**184**							41.6
Ct3BOT1	107	159			**976**		196	147			
Ct3BOT2						**674**					
Ct3BOT3	98.6	126				199	**1036**	127			
Ct3BOT4	107	261			136		137	**958**			
Ct3BOT5									**870**		
Ct3BOT6										**483**	
Ct3BOT7				53.9							**557**

Supplementary Table 4: Expression of Ct3, Ct4, Ct61 or KHC ABA and BOT genes during the root colonization. The values represent fpkm. This value was calculated based on Ct3, Ct4, Ct61 or KHC draft genome assemblies.

Ct3

		ABA1	*ABA1*	*ABA2*	*ABA3*	*ABA4*	*BOT1*	*BOT2*	*BOT3*	*BOT4*	*BOT5*	*BOT6*	*BOT7*
	Normal Pi	125.251	91.7376	352.335	128.089	9.22808	412.383	385.366	135.292	295.088	237.207	473.298	2760.42
10 dpi	Low Pi	137.857	81.0386	422.115	128.999	0	302.254	338.355	105.574	243.581	185.434	472.06	2783.61
	Normal Pi	0	0	0	0	0	9.2847	0	0	0	0	8.11209	709.583
24 dpi	Low Pi	12.046	19.0386	0	0	0	0	6.19581	0	5.11354	0	16.4728	392.748

Ct4

		ABA1	*ABA2*	*ABA3*	*ABA4*	*BOT1*	*BOT2*	*BOT3*	*BOT4*	*BOT5*	*BOT6*	*BOT7*
	Normal Pi		0	0	0	1.1704	8.95731		1.38108	0	11.1849	220
10 dpi	Low Pi		0	0	0	0.4132	0		12.3495	0	6.69306	216.865
	Normal Pi		0	22	0	0	10.9126		0	0	33.5001	1240.51
24 dpi	Low Pi		5.54179	4	0	3.80622	12.2368		15.7535	3.07394	0	1058.05

Ct61

		ABA1	*ABA2*	*ABA2*	*ABA3*	*ABA4*	*BOT1*	*BOT2*	*BOT3*	*BOT4*	*BOT5*	*BOT6*	*BOT7*
	Normal Pi	1.85582	2.13089	5.47731	1.56631		9.38142	14.1809	9.71836	5.69527	12.8488		618.987
10 dpi	Low Pi	0	0.697352	0	2.72031		7.92429	7.38662	8.63708	12.6158	6.07758		558.398
	Normal Pi	0	0	0	0		0	9.14703	0	0	0		851.557
24 dpi	Low Pi	0	0	13.9356	8.96509		38.1794	63.3038	19.5615	42.5972	9.43841		1146.65

KHC

		ABA1	*ABA1*	*ABA2*	*ABA3*	*ABA4*	*BOT1*	*BOT2*	*BOT3*	*BOT4*	*BOT5*	*BOT6*	*BOT7*
	Normal Pi	0	0	0	0	11.0101							
10 dpi	Low Pi	0	0	4.49654	0	0							
	Normal Pi	0	0	0	0	28.8942							
24 dpi	Low Pi	0	0	2.15742	0	8.51124							

a) Note that automatically annotated *Ct3ABA1*, *Ct61ABA2* and *KHCABA1* genes were separated into two.

*Ct*ABA genes used in Fig. 4 and Supplementary Fig. 4 were re-predicted using RNAseq-derived reads. The reannotated amino acid sequences are available from Supplementary Fig. 4o.

Supplementary Table 5: Primers used in this study

Gene/Region	Gene_Name or Primer Name	Forward	Reverse	Use
AT3G18780	*ATACT2*	ACCTTGCTGGACGTGACCTTACTGAT	GTTGTCTCGTGGATTCCAGCAGCTT	qRT-PCR
AT5G52310	*RD29A*	CAAAGGTGTTTCCTGTCGTGTC	AATCGGTACATCTCTTTTCTCTTCC	qRT-PCR
AT1G05100	*MAPKKK18*	AAGCGGCGCGTGGAGAGAGA	GCTGTCCATCTCTCCGTCGC	qRT-PCR
AT5G03545	*AT4*	GTCTTTTGTTATCCCTCGTTTGGG	ACCGGAAACAAAGTAAACACGG	qRT-PCR
CTACT	*CtACT*	GAGTCCTTCTGGCCCATAC	GAGTCCTTCTGGCCCATAC	qRT-PCR
CT3ABA3	*Ct3ABA3*	ACCTCGTGAGCGCGATACATGGTA	GTTGAGGACACTGTCGCCCAAT	qRT-PCR
CT3BOT5	*Ct3BOT5*	ACGAGGTGTTGCCCGTACACTT	GGAAAGGTCGACGTGGGAGAACTT	qRT-PCR
CiABA3	*CiABA3*	TCATGCGGCTGGTACCAACAA	CTTTTGATTCCTGGGCTTGC	qRT-PCR
CiBOT5	*CiBOT5*	ACCACTTTTCTTCTCACTCGTG	TGATGATGGATGTGCGTACAATCG	qRT-PCR
Ct3ABA2	Ct3ABA2_infu_5F	TATCGATACCGTCGACTATGCAACTAGAGACCACGC	ACCAGTTAACGTCGACCGTCACAATTAAACTAATTAGG	Knockout
Ct3ABA2	Ct3ABA2_infu_3F	ATCAGTTAACGTCGACATTCAACGGGTTATTTTCGAAG	CCCCCTCGAGGTCGACCAGAACCCTCAAATCGAGAAG	Knockout
Ct3ABA3	Ct3ABA3_infu_5F	TATCGATACCGTCGACCATTCGACCTGATCCAGCCCAT	ACCAGTTAACGTCGACCCTGGCAGGTTCTTTGGCGTG	Knockout
Ct3ABA3	Ct3ABA3_infu_3F	ATCAGTTAACGTCGACGAGCCATGGGATAAAGATAACTG	CCCCCTCGAGGTCGACACCGCACGCTCAACTTCATCGA	Knockout
Ct3BOT1	Ct3BOT1_infu_5F	TATCGATACCGTCGACGAGAAGCTGTGCGAAGTAGCGCT	ACCAGTTAACGTCGACCGTAGGACGTGTGCTGCGGGCTTG	Knockout
Ct3BOT1	Ct3BOT1_infu_3F	ATCAGTTAACGTCGACTGGATGATGGATTACGGACACAC	CCCCCTCGAGGTCGACAGACGGACCGTGTAAGCAAGA	Knockout
Ct3BOT3	Ct3BOT3_infu_5F	TATCGATACCGTCGACAGTTCCTCCACGGGCTCGATGATA	ACCAGTTAACGTCGACGATAACGTCACCGACTGTAAAG	Knockout
Ct3BOT3	Ct3BOT3_infu_3F	ATCAGTTAACGTCGACAAACAGACTCCGGTTGGTATG	CCCCCTCGAGGTCGACGCTGAGGTTCGGGAAGCCTTCAAT	Knockout
Ct3BOT5	Ct3BOT5_infu_5F	TATCGATACCGTCGACGCTTGCATACCGCTAGACACCT	ACCAGTTAACGTCGACGTTGTGTATGGGTAGCTGCTGCG	Knockout
Ct3BOT5	Ct3BOT5_infu_3F	ATCAGTTAACGTCGACTGTCGTGGTTGAGGCCTAGTCG	CCCCCTCGAGGTCGACTCCATTACCTTTATCTCGGGTC	Knockout
Ct3BOT5	Ct3BOT5_infu_Com	CGGCCCATGGTCTAGAAACGCCCGACAAGTCCTTCTT	GCTTGTTAACGAATTCAGGCGCTTTTACCCACCTCTA	Complementation
