# Supplementary material for: A fungal sesquiterpene biosynthesis gene cluster critical for mutualist-pathogen transition in Colletotrichum tofieldiae
Source: Nat Commun. 2023 Sep 6;14:5288. doi: 10.1038/s41467-023-40867-w (PMC10482981; doi:10.1038/s41467-023-40867-w)
Supplement: Supplementary file 3 — Description of Additional Supplementary Files [file 41467_2023_40867_MOESM3_ESM.pdf]

Description of Additional Supplementary Files:

Supplementary Data 1: Accessions of the 1,509 single-copy orthologous protein sequences used for phylogenetic analysis (Fig. 2b).

Supplementary Data 2: List of A. thaliana genes whose expression was significantly influenced between sample_1 and sample_2 (q (FDR) < 0.05) at 10 days post-inoculation (dpi). P = normal Pi. pp = low Pi. Treatment: Mock, Ct61, Ct4, Ct3, or KHC.

Supplementary Data 3: Gene ontology analyses for 758 A. thaliana genes specifically and significantly upregulated during root colonization by pathogenic Ct3 compared with beneficial Ct61 and Ct4 at 10 dpi (log2FC >1, q (FDR) < 0.05). Genes were separated into three different clusters by k-means.

Supplementary Data 4: List of Ct3 genes significantly influenced during A. thaliana root colonization between 10 dpi and 24 dpi (q (FDR) < 0.05). P = normal Pi. mp = low Pi. e = 10 dpi. L = 24 dpi.

Supplementary Data 5: List of Ct3 92 genes whose genes was co-regulated with Ct3 ABA or BOT genes. This is related to Supplementary Table 6.

Supplementary Data 6: ABA (1-4) or BOT (1-7) homologous genes used as the query of BLASTP search against GenBank NR database.

Supplementary Data 7: Amino acid sequences used in Fig. 4, 8, Supplementary Figs. 4, 8.

Supplementary Data 8: List of Ct3 genes whose expression was significantly influenced between sample_1 and sample_2 (q (FDR) < 0.05) at 10 days post-inoculation (dpi) under low Pi. Treatment: Ct3, Ct3Δaba2, Ct3Δaba3, Ct3Δbot5.

Supplementary Data 9: List of Ct3 92 genes described in Supplementary Table 7. The expression profiles of each Ct3 gene during root colonization with Ct3WT, Ct3Δaba2, Ct3Δaba3, or Ct3Δbot5 were described.

Supplementary Data 10: List of A. thaliana genes whose expression was significantly influenced between sample_1 and sample_2 (q (FDR) < 0.05) at 10 days post-inoculation (dpi) under low Pi. Treatment: Mock, Ct3, Ct3Δaba2, Ct3Δaba3, Ct3Δbot5, or Ct4.

Supplementary Data 11: A total of 288 A. thaliana genes were significantly induced during root colonization with Ct3WT compared with Ct3Δaba2, Ct3Δaba3, Ct3Δbot5, and Ct4 (log2FC >1, q (FDR) < 0.05).

Supplementary Data 12: 375 A. thaliana genes were significantly suppressed after inoculation with Ct3WT compared to those with Ct3Δaba2, Ct3Δaba3, Ct3Δbot5, and Ct4 (log2FC < 0.05).

Supplementary Movie 1: z-stack of Ct3 expressing cytoplasmic GFP (green) and root cells of A. thaliana expressing PIP2A-mCherry (magenta) shown in Fig. 1e. White arrowheads indicate the Ct3 hypha penetrating a root epidermal cell surrounded by PIP2A-mCherry-labeled host membranes.

Supplementary Movie 2: z-stack of Ct4 expressing cytoplasmic GFP (green) and root cells of A. thaliana expressing PIP2A-mCherry (magenta) shown in Fig. 1f. White arrowheads indicate the Ct4 hypha penetrating a root epidermal cell surrounded by PIP2A-mCherry-labeled host membranes.
